# Element-specific contributions to improved magnetic heating of theranostic CoFe_2_O_4_ nanoparticles decorated with Pd

**DOI:** 10.1038/s41598-021-95189-y

**Published:** 2021-08-04

**Authors:** S. Fatemeh Shams, Detlef Schmitz, Alevtina Smekhova, Mohammad Reza Ghazanfari, Margret Giesen, Eugen Weschke, Kai Chen, Chen Luo, Florin Radu, Carolin Schmitz-Antoniak

**Affiliations:** 1 Peter-Grünberg-Institut (PGI-6), Forschungszentrum Jülich, 52425 Jülich, Germany; 2 Helmholtz-Zentrum Berlin für Materialien und Energie, 14109 Berlin, Germany; 3 Institute of Chemistry and Biochemistry, Freie Universität Berlin, 14195 Berlin, Germany; 4 Present Address: Helmholtz-Zentrum Berlin für Materialien und Energie, 12489 Berlin, Germany; 5 Present Address: Technische Hochschule Wildau, Hochschulring 1, 15745 Wildau, Germany

**Keywords:** Physics, Condensed-matter physics, Magnetic properties and materials

## Abstract

Decoration with Pd clusters increases the magnetic heating ability of cobalt ferrite (CFO) nanoparticles by a factor of two. The origin of this previous finding is unraveled by element-specific X-ray absorption spectroscopy (XAS) and magnetic circular dichroism (XMCD) combined with atomic multiplet simulations and density functional theory (DFT) calculations. While the comparison of XAS spectra with atomic multiplet simulations show that the inversion degree is not affected by Pd decoration and, thus, can be excluded as a reason for the improved heating performance, XMCD reveals two interrelated responsible sources: significantly larger Fe and Co magnetic moments verify an increased total magnetization which enhances the magnetic heating ability. This is accompanied by a remarkable change in the field-dependent magnetization particularly for Co ions which exhibit an increased low-field susceptibility and a reduced spin canting behavior in higher magnetic fields. Using DFT calculations, these findings are explained by reduced superexchange between ions on octahedral lattice sites via oxygen in close vicinity of Pd, which reinforces the dominating antiparallel superexchange interaction between ions on octahedral and tetrahedral lattice sites and thus reduces spin canting. The influence of the delocalized nature of Pd 4d electrons on the neighboring ions is discussed and the conclusions are illustrated with spin density isosurfaces of the involved ions. The presented results pave the way to design nanohybrids with tailored electronic structure and magnetic properties.

## Introduction

Besides a possible use in spintronics, a large field of applications for cobalt ferrite (CFO) and other ferrites at the nanoscale can be found in biomedicine: superparamagnetic nanoparticles of CFO and magnetite phases exhibit physicochemical stability and are used e.g. as contrast agents in magnetic resonance imaging (MRI), carriers for smart drug delivery (SDD) and for local heating in magnetic fluid hyperthermia (MFH) as reviewed in Ref.^1^. Their functionalities can be synergistically improved in hybrid nanostructures^2–4^ leading to elaborate designs of theranostic nano-agents with both, therapeutic and diagnostic capabilities^5–7^. Since colloidal heterodimers can be efficiently utilized for a wide range of bioapplications, we had already introduced Pd decorated CFO nanoparticles for potential optical and magnetic hyperthermia applications^8^. Although some noble metals like gold and silver provide plasmonic behavior as a significant mechanism of photothermal therapy, they mostly exhibit diamagnetic properties that could lead to substantially reduced magnetization. However, metallic palladium does not only show substantial plasmonic properties but also Stoner-enhanced paramagnetism. Therefore, it might be considered as a suitable candidate for the simultaneous plasmonic-magnetic hyperthermia bioapplications. Moreover, Pd decorations could improve the performance in photothermal therapeutic applications, the biocompatibility of CFO clusters, and the heat transfer efficiency in magnetic hyperthermia^8^. In particular, it has been recently shown that CFO-Pd heterodimers, i.e. CFO nanoparticles decorated with Pd nanoparticles, exhibit specific absorption rates in suspension of up to 66–68 W/g (normalized to the mass of magnetic material) that outperform commercially available ferrofluids used for magnetic heating by about a factor of two^8^.

CFO ﻿is a ferrimagnetic oxide that crystallizes in a cubic inverse spinel structure with sizeable cation disorder. Its chemical formula can be written as

$${\left({\text{Co}}_{1-i}^{2+}{\text{Fe}}_{i}^{3+}\right)}_{Td}{\left({\text{Co}}_{i}^{2+}{\text{Fe}}_{2-i}^{3+}\right)}_{Oh}{\text{O}}_{4}^{2-}$$

where *T*_*d*_ denotes lattice sites tetrahedrally coordinated by oxygen ions, *O*_*h*_ octahedrally coordinated lattice sites, and *i* accounts for the degree of inversion, a metric of cation disorder. The perfect inverse spinel is characterized by *i* = 1. In this case, all Co^2+^ ions occupy octahedral sites and the Fe^3+^ ions are equally distributed on octahedral and tetrahedral sites. In a simple two-sublattices model, a dominant intersublattice antiferromagnetic superexchange between ions on tetrahedral and octahedral sites leads to a ferrimagnetic ground state in which the magnetic moments carried by the Fe ions cancel out. Consequently, the net magnetic moment is caused by the magnetic moment of Co ions, i.e. 3 µ_B_ per formula unit if the orbital magnetism is neglected. In this simple model, the special case of *i* = 0 would be connected to a net saturation magnetic moment of 7µ_B_ per formula unit caused by the antiferromagnetic coupling between Co^2+^ (3d^7^, 3µ_B_ spin-only) on tetrahedral sites and the two Fe^3+^ ions (each 5 µ_B_) on octahedral sites. For intermediate degrees of inversion, the magnetic moment would be between 3 and 7 µ_B_ in saturation.

Beyond this simple model, in real CFO systems, magnetic properties like saturation magnetization, magnetic anisotropy, and temperature-dependent magnetization exhibit a rather complex behavior with non-collinear magnetic moments and depend on e.g. the crystallite size^9,10^, degree of inversion^11–13^, and antiphase boundaries^11,14^. Across an antiphase boundary between cation lattices, the spin couplings are reversed leading to oppositely magnetized regions while the oxygen crystal structure remains undisturbed. These displacements are not observable in conventional structural analyses but create strong 180° antiferromagnetic exchange coupling between octahedrally coordinated cations associated with the creation of antiparallel aligned magnetic domains yielding a significantly reduced net magnetization^14^.

In general, the competition between different superexchange couplings, magnetostatic (Zeeman) energy, and magnetic anisotropies leads to ion- and site-specific spin canting effects that reduce the observed magnetization in experiments. For instance, with Mössbauer spectroscopy, a non-collinear spin structure has been revealed for ferrite nanoparticles that may occur preferentially for ions on *O*_*h*_ sites^15^ or extend to both *T*_*d*_ and *O*_*h*_ sites^16^.

In the theoretical work of Ansari et al.^17^, the superexchange parameters for nanoscale CFO with structural distortions and cation disorders have been calculated from DFT in terms of the Heisenberg model: while for *i* = 1, the superexchange between *T*_*d*_ and *O*_*h*_ sites is dominating for Fe and Co ions, for *i* < 1 particularly the antiferromagnetic Co(*O*_*h*_)–Co(*O*_*h*_) superexchange increases and may even overcome the superexchange coupling with ions on *T*_*d*_ sites. Furthermore, the Co(*O*_*h*_)–Co(*T*_*d*_) exchange coupling constant is smaller by a factor two compared to Co(*O*_*h*_)–Fe(*T*_*d*_), which reduces the intersublattice superexchange and yields a reduced Curie temperature for *i* < 1 according to mean-field theory.

Typical degrees of inversion have been found for CFO in the range of *i* = 0.68–0.8^12,18,19^. And in agreement with the theoretical results^17^ mentioned above, Sawatzky et al. concluded from Mössbauer studies^13^ that the replacement of an Fe^3+^ ion by a Co^2+^ ion at a tetrahedral lattice site reduces the superexchange significantly and leads to a more rapidly decreasing magnetization when going from low temperatures to room temperature. Hence the complex interplay between magnetism, structure and cation order makes it necessary to carefully characterize CFO samples, particularly when aiming at specific applications at room temperature and above.

Coming back to the doubling of the specific absorption rates of suspended CFO nanoparticles used for magnetic heating as mentioned above, the size, cation disorder and magnetic anisotropy have already been excluded as reasons for the enhanced magnetic heating upon decoration with Pd^8^. Moreover, a potentially induced magnetization of Pd clusters, which could be expected because of the Stoner-enhanced magnetic polarizability of Pd, cannot explain the observed changes in the magnetic response either, because the amount of Pd (< 2 wt%) is too small.

Therefore, we focus in this work on the influence of Pd on the magnetism of Fe and Co ions in CFO to unravel the microscopic origin behind the macroscopic improvement of magnetic properties. For this purpose, X-ray absorption spectroscopy and its magnetic dichroisms have been used to (1) quantify the cation disorder by comparison with atomic multiplet calculations, (2) determine effective spin and orbital moments of Fe and Co ions for bare CFO nanoparticles and CFO-Pd heterodimers, and (3) measure the field-dependent magnetization element-specifically. In addition, the magnetic properties found in the experiments are explained with DFT calculations by assessing the competing magnetic interactions and analyzing the spin density isosurfaces.

## Results and discussion

The samples have been investigated with regard to their size (small and large), composition (bare and decorated), degree of inversion, Pd content and mass magnetization. The resulting values are summarized in Table 1.

**Table 1:** Sample labelling, composition, mean crystallite diameter, degree of inversion from XANES and XMCD, Pd content (where applicable), and mass magnetization from VSM in a magnetic field of 6 T at temperatures of 8 K and 300 K, respectively.

Sample	Composition	CFO crystallite diameter (nm)	Degree of inversion				Pd (wt%)	Mass magnetization M(6 T) (Am^2^/kg)	
			300 K		5 K				
			Fe	Co	Fe	Co		300 K^8^	8 K
S	bare CFO	5.1 ± 5%	0.79	0.72	0.80	0.79	–	8 ± 1	15 ± 2
S-Pd	Pd decorated CFO	5.1 ± 5%	0.85	0.75	0.82	0.80	1.61	33 ± 5	48 ± 7
L	bare CFO	17.8 ± 5%	0.84	0.72	0.83	0.79	–	15 ± 2	30 ± 5
L-Pd	Pd decorated CFO	17.8 ± 5%	0.83	0.80	0.83	0.79	1.31	30 ± 5	47 ± 7

### Composition, structure, topography, and magnetization

For bare CFO samples, the inductively coupled plasma atomic emission spectroscopy (ICP-OES) investigations revealed mass ratios m(Fe)/m(Co) in the range of 1.96–1.98 corresponding to a slightly Fe-rich composition with 2.06–2.09 Fe ions per Co ion. To ensure charge neutrality and stability of the synthesized structures, cation vacancies arise^20–22^. Note that these vacancies can facilitate the possibility to attach Pd ions in the second step of the synthesis. Formally, the small Fe enrichment increases the magnetic moment of the CFO compound compared to the stoichiometric CoFe_2_O_4_ while maintaining the large magnetic anisotropy.

X-ray diffraction (XRD) data (Supplementary Figure S1) of bare CFO samples exhibit merely the peaks of cobalt ferrite crystallized in the inverse spinel structure that confirm the absence of any undesired intermediate phases such as cobalt oxide (CoO). According to Rietveld structure refinements, the crystallite sizes are equal to (5.1 ± 5%) nm and (17.8 ± 5%) nm for small (S) and large (L) CFO nanoparticles, respectively, in agreement with inherently larger hydrodynamic diameters determined from dynamic light scattering (Supplementary Figure S1). Furthermore, the crystallinity degrees of the CoFe_2_O_4_ phase are about 82% and 85% in small and large CFO nanoparticles, respectively. In addition, by Rietveld refinement the cation disorder was found to be slightly larger for the small CFO nanoparticles. More details about cations distribution and other structural parameters like lattice parameters of bare CFO nanoparticles as well as the colloidal stability properties are reported in our previous publications^23,24^. The results show that both bare samples have similar compositional and structural properties and differ almost solely in their particle diameter.

After Pd decoration, the composition was analyzed again with ICP–OES and Pd was detected in both CFO-Pd hybrid samples, namely 1.611 and 1.311 wt% for small and large CFO nanoparticles, respectively, with an uncertainty of 0.2%. The difference in Pd decoration for both samples could be explained by variations in the surface fraction serving as possible attachment sites for the ultrasmall Pd clusters. The ratio of Fe to Co was found to be identical to the bare CFO nanoparticles within experimental uncertainties.

Microscopic analyses of bare and Pd decorated CFO nanoparticles show the homogeneous nanometric size of particles with uniform, nearly spherical shapes. In addition, 3D topographies from atomic force microscopy (AFM) images of prepared samples for X-ray absorption near edge structure (XANES) and XMCD measurements on highly-oriented pyrolytic graphite (HOPG) and silicon wafers visualize the size- and shape-uniformity of the nanoparticles. Corresponding AFM topography micrographs of bare CFO samples taken in amplitude and phase mode and a 3D reconstruction are provided in Supplementary Figure S2.

Magnetometry data reveal a substantial impact of Pd decoration on the magnetic properties, in particular an increase of the magnetization in an external field of 6 T at 300 K (Table 1) by a factor of about 4 (2) for the small (large) CFO nanoparticles compared to the bare ones and changes in the magnetic susceptibility^8^. Admittedly, the high-field magnetization values for both bare samples are lower than some reported values in the literature which could be due to the presence of spin-disorder at the surface, different degrees of cation inversion^13^, anti-phase boundaries^14^, aging effects^25^, chelating agents especially citric acid^26^, and agent type for pH value of reaction medium^27^. Low-temperature magnetometry data and magnetometry data at 300 K are shown in Supplementary Figures S3, S4, respectively, and are discussed in the Supplementary Information.

### Cation disorder from element-specific XAS

Electronic structure and magnetic properties such as total magnetization and magnetic anisotropy in ferrites significantly depend on the degree of inversion. In order to distinguish between the influence of Pd decoration and possible differences in the cation disorder, the fine structure in XANES and XMCD at both Co L_3,2_ and Fe L_3,2_ edges have been investigated and compared to atomic multiplet calculations. As presented below, the results confirm the cation disorder obtained from Rietveld refinement analysis of XRD data^24^.

**Figure 1:**
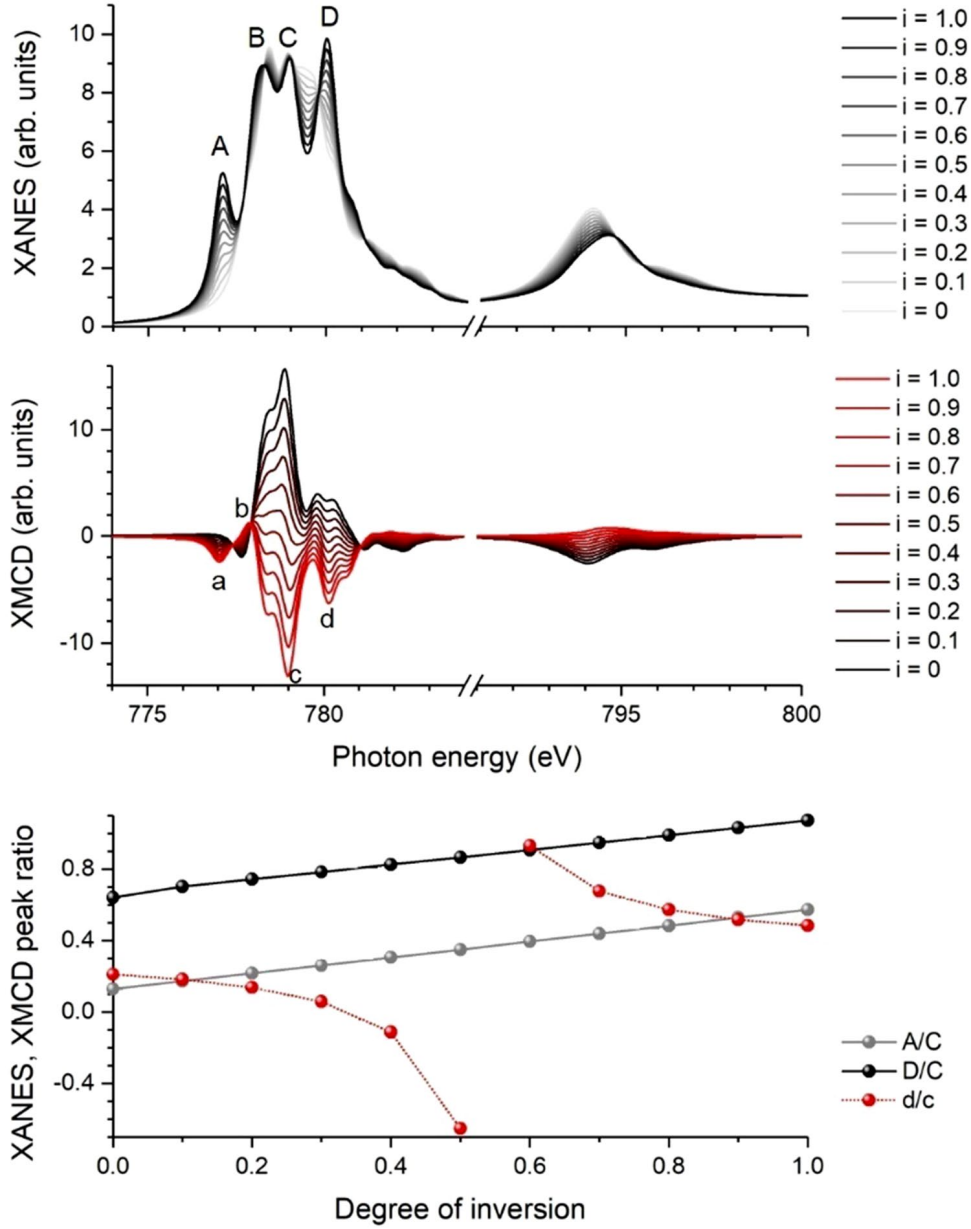
Influence of cation disorder on spectral features of Co. Simulated spectral changes with degree of inversion in CFO for XANES (top) and XMCD (center) at the Co L_3,2_ absorption edges as well as selected peak ratios as a function of inversion degree (bottom).

In Fig. 1, simulated spectra from atomic multiplet calculations at the Co L_3,2_ edges are presented for different degrees of inversion. For XANES at the Co L_3_ absorption edge (top panel) changes in the relative peak ratios are visible. For a perfect inverse spinel structure (*i* = 1), peak A is clearly visible and decreases with decreasing degree of inversion. The same trend can be observed for peak D. The amplitude of peak B exhibits only small changes and an energy shift of the peak position is visible. Amplitude and energy position of peak C remains almost unaffected by the cation disorder and was chosen as a reference amplitude. Consequently, the peak ratios A/C and D/C were used to determine the degree of inversion. Their approximately linear dependences on the degree of inversion are shown in the bottom panel of Fig. 1. The simulated XMCD spectra are shown in the central panel of Fig. 1. The most striking change is the change of sign of the main peak c with cation disorder between *i* = 0.5 and *i* = 0.6 because of the ferrimagnetic order in CFO. Negative values of peak c indicate *i* > 0.5, i.e. the structure is closer to the inverse spinel. For peak d, a change of sign is observed for smaller values between *i* = 0.3 and *i* = 0.4. The changes of peaks a and b are clearly visible as well. Since their absolute values are rather small, peaks c and d seem to be more suitable for the analysis of cation disorder and the ratio d/c was chosen here to estimate the degree of inversion from experimental XMCD spectra. The advantage of the peak ratio compared to the single amplitudes is that it is less sensitive to possible technical artifacts and differences in the magnetic moments between experiment and theory.

**Figure 2:**
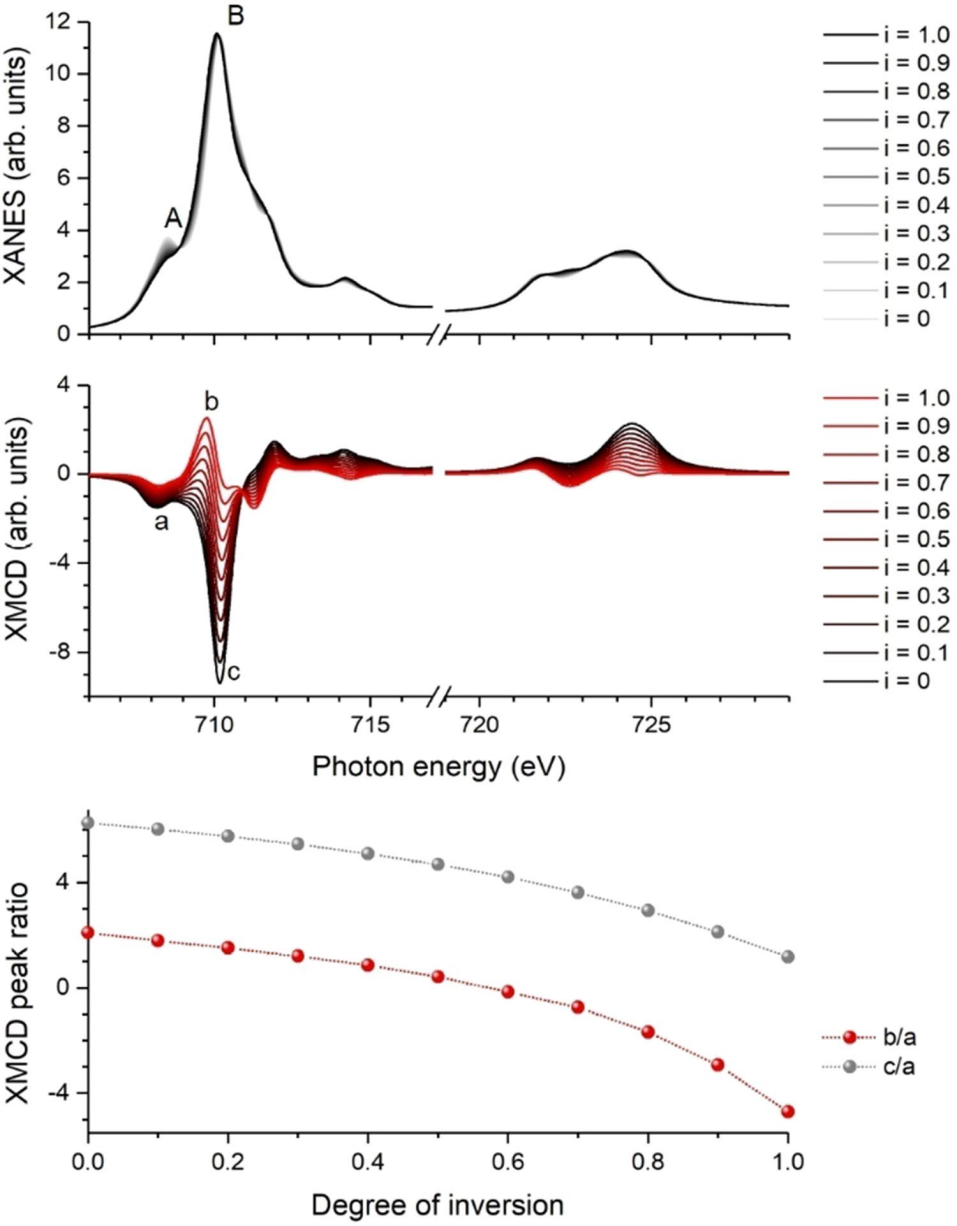
Influence of cation disorder on spectral feature of Fe. Simulated spectral changes with degree of inversion in CFO for XANES (top) and XMCD (center) at the Fe L_3,2_ absorption edges as well as selected peak ratios as a function of inversion degree (bottom).

The simulated dependences of the spectral shapes of XANES and XMCD on the degree of inversion at the Fe L_3,2_ absorption edges are shown in Fig. 2. While the XANES peak ratio A/B shows only slight changes, the XMCD peak ratios b/a and c/a change significantly. The ratio b/a even changes its sign between a degree of inversion of *i* = 0.6 and *i* = 0.7. However, due to deviations of the Fe charge from its nominal value of + 3, the spectral contributions of Fe ions on octahedral and tetrahedral sites may be closer in energy yielding smaller absolute values of the amplitudes b and c. Therefore, the ratio b/a underestimates the degree of inversion while c/a overestimates the degree of inversion. For a more reliable analysis, both peak ratios should be considered. Note that in general the accuracy of inversion degree values extracted from XMCD spectra is lower compared to an analysis of XANES data because of possible site-specific spin canting effects or magnetically “dead” surface layers in combination with a radial cation disorder gradient.

Simulated XMLD spectra for different degrees of inversion are presented in the Supplementary Information in Fig. S7. It cannot be used here to analyze the cation disorder because the anisotropic natural linear dichroism^28^, as present in the simulations, cancels out in the experiment due to a random distribution of the nanoparticles’ crystal orientations.

**Figure 3:**
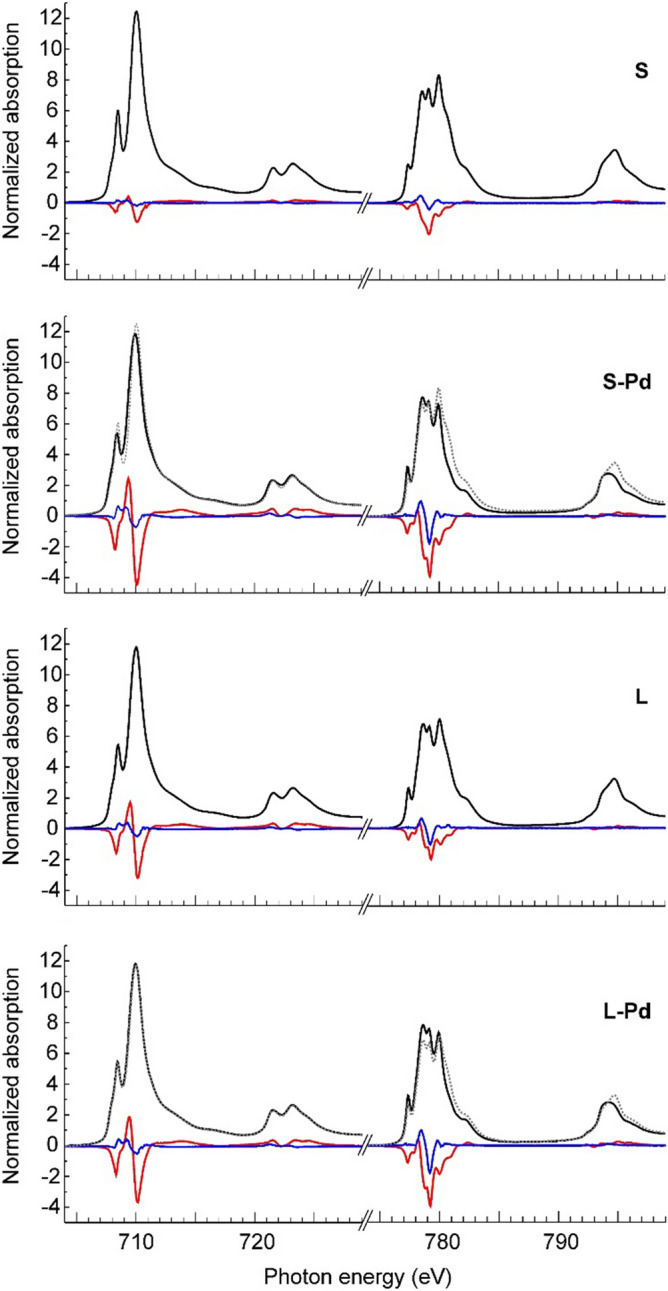
Low-temperature X-ray absorption spectra of CFO and CFO-Pd nanoparticles. XANES (black lines), XMCD (red lines) and XMLD (blue lines) spectra in a magnetic field of 6 T at a temperature of 5 K at the *L*_*3,2*_ absorption edges of iron and cobalt cations in 5.1 nm small CFO nanoparticles, bare (S) and Pd decorated (S-Pd), and large 17.8 nm CFO nanoparticles, bare (L) and Pd decorated (L-Pd). XANES of bare CFO nanoparticles after Pd decoration (dotted lines).

**Figure 4:**
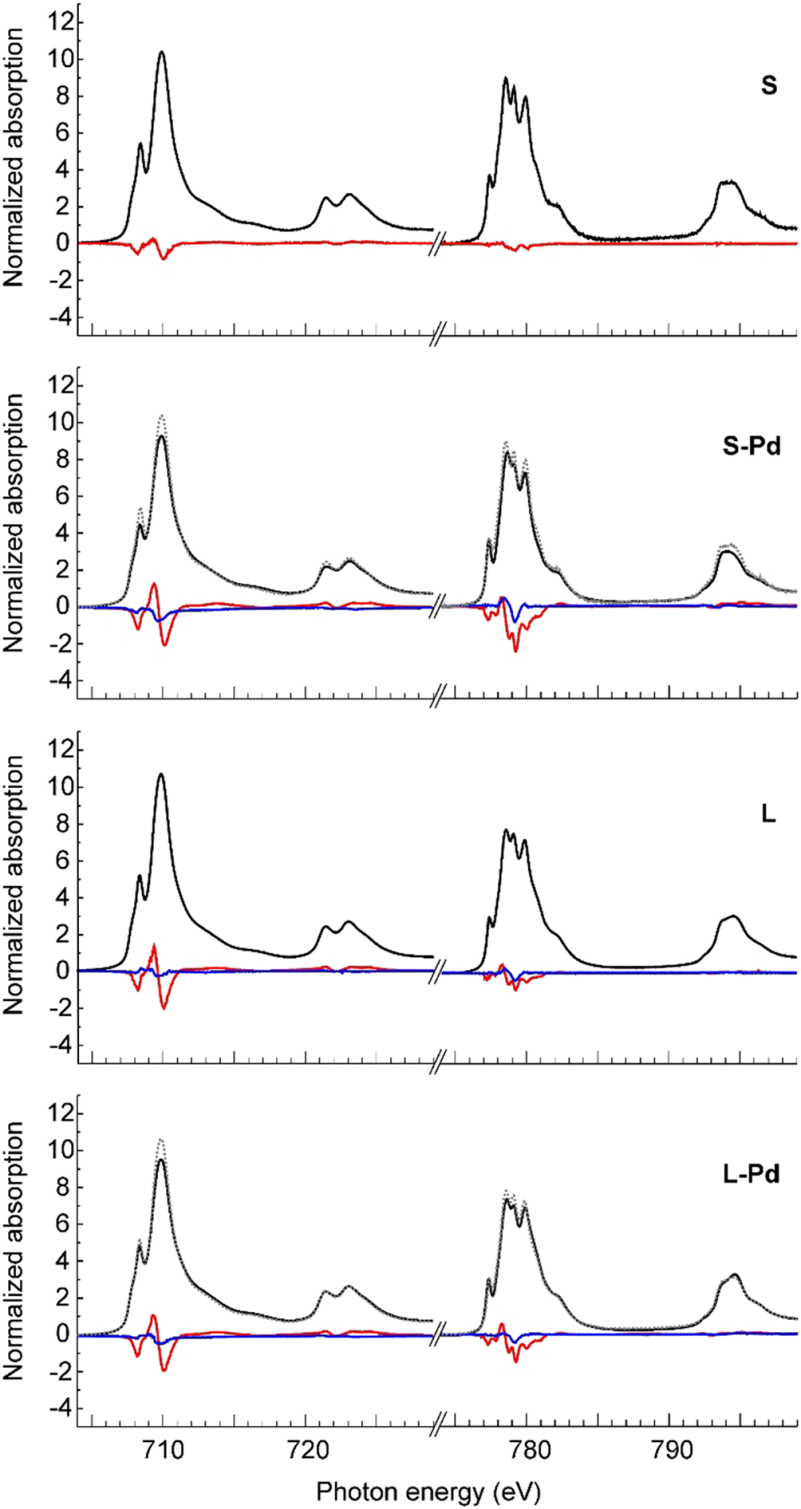
Room-temperature X-ray absorption spectra of CFO and CFO-Pd nanoparticles. XANES (black lines), XMCD (red lines) and XMLD (blue lines) spectra in a magnetic field of 6 T at a temperature of 300 K at the *L*_*3,2*_ absorption edges of iron and cobalt cations in 5.1 nm small CFO nanoparticles, bare (S) and Pd decorated (S-Pd), and large 17.8 nm CFO nanoparticles, bare (L) and Pd decorated (L-Pd).

The experimental XANES, XMCD, and XMLD spectra are presented in Fig. 3 for *T* = 5 K and in Fig. 4 for *T* = 300 K. XANES data are shown as black lines, XMCD as red lines, and XMLD as blue lines. For comparison, XANES of bare CFO nanoparticles is also shown together with the spectra obtained after Pd decoration as dotted lines. In the low temperature spectra, Peak D in the Co L_3_ XANES exhibits a strongly enhanced amplitude compared to the data measured at 300 K pointing towards a charge localization at low temperatures. Further temperature dependent studies are needed to investigate this effect in more detail, which is beyond the scope of this paper.

The degree of inversion, separately determined from the five peak ratios mentioned above for Co and Fe, were found to range between 0.79 and 0.85 and are summarized in Table 1 for *T* = 300 K and *T* = 5 K. By averaging the results obtained from different spectral features, the uncertainty reduces to about 0.05. However, relative changes can be observed with a higher accuracy of about 0.02.

In accordance with results from Rietveld refinement analysis of diffraction data, the degree of inversion is similar for all samples. A larger degree of inversion for Fe ions compared to Co ions in the slightly Fe-rich CFO nanoparticles indicates the presence of additional vacancies at octahedral lattice sites as expected to maintain charge neutrality. For both, small and large CFO nanoparticles, the degree of inversion is slightly increased upon decoration with Pd. However, the changes are close to the experimental uncertainty. From the simple two-sublattices model with collinear spins, the increased degree of inversion determines a decrease in the total saturation magnetic moment of CFO. Since magnetometry measurements revealed that both, increasing size and Pd decoration, lead to higher values of mass magnetization (uncertainties 5%), the slight changes in cation disorder can be ruled out as the leading direct mechanism for these differences in magnetism upon decoration with Pd.

### Element-specific magnetic moments from XMCD

**Figure 5:**
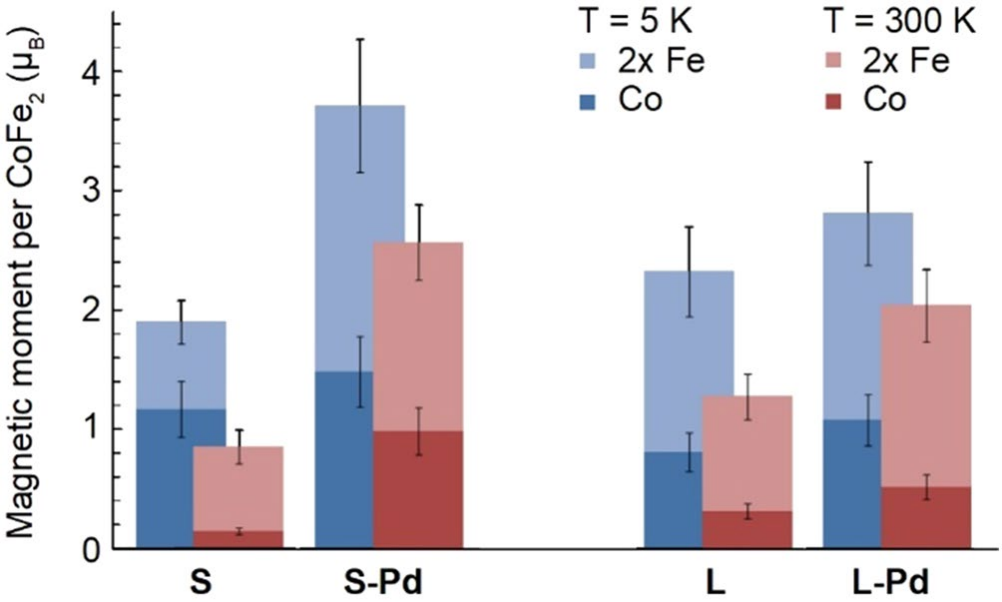
Element-specific magnetic moments. Element-specific contributions to the total magnetic moment per formula unit CoFe_2_ (neglecting any oxygen contribution) for 5.1 nm small CFO nanoparticles, bare (S) and Pd decorated (S-Pd), and large 17.8 nm CFO nanoparticles, bare (L) and Pd decorated (L-Pd) in magnetic fields of 6 T at temperatures of *T* = 5 K (blue) or *T* = 300 K (red).

Element-specific effective spin and orbital moments were extracted from XANES and XMCD data by a sum-rules^29–31^ based analysis according to:

1 $$m_{S}^{eff} = - \, \left( {3p - 2q} \right) n_{h} /r$$

2 $$m_{L} = - 2qn_{h} /3r$$

3 $$m_{L} /m_{S}^{eff} = 2q/\left( {9p - 6q} \right)$$

where *p* denotes the integral of XMCD over the L_3_ absorption edge, *q* denotes the integral of XMCD over both L_3_ and L_2_ absorption edges, and *r* denotes the integrated averaged 3d-XANES after subtraction of a two step-like function accounting for electron excitation into higher unoccupied states or the continuum. The number of unoccupied final states *n*_*h*_ (*n*_*h*_ = 5 for Fe^3+^ and *n*_*h*_ = 3 for Co^2+^ free ions) should be proportional to *r* and has been carefully monitored. Details about the data treatment, integral values as well as experimental and sum rule related uncertainties can be found in the Supplementary Information. Effective spin and orbital moments are summarized in Tables 2 and 3 for *T* = 5 K and *T* = 300 K, respectively, and shown in Fig. 5 to depict the changes. Note that in a magnetic field of 6 T the samples were not magnetically saturated. Experimental uncertainties and possible limitations of the sum-rule accuracy lead to a total uncertainty of about 20% for the absolute values of effective spin moments and 10% for the orbital moments. Relative changes can be observed with much higher accuracy (estimated 5% uncertainty for effective spin and orbital moments).

**Table 2:** Low-temperature element-specific magnetic moments of Fe and Co cations (*µ*_0_*H* = 6 T, *T* = 5 K) in 5.1 nm small CFO nanoparticles, bare (S) and Pd decorated (S-Pd), and large 17.8 nm CFO nanoparticles, bare (L) and Pd decorated (L-Pd).

Sample	Fe magnetic moments				Co magnetic moment				Magnetic moment CoFe_2_ (µ_B_)
	*m*_*S*_^*eff*^ (µ_B_)	*m*_*L*_ (µ_B_)	*m*_*L*_*/m*_*S*_^*eff*^ (%)	*m*_*tot*_ (µ_B_)	*m*_*S*_^*eff*^ (µ_B_)	*m*_*L*_ (µ_B_)	*m*_*L*_*/m*_*S*_^*eff*^ (%)	*m*_*tot*_ (µ_B_)	
S	0.36	0.01	2	0.37	0.80	0.37	46	1.17	1.91
S-Pd	1.11	0.00	0	1.11	1.05	0.43	41	1.48	3.70
L	0.76	0.00	0	0.76	0.54	0.27	48	0.81	2.33
L-Pd	0.87	0.00	0	0.87	0.76	0.32	42	1.08	2.82

Uncertainties are 20% for the effective spin moments, 10% for the orbital moment, and 10% for the ratio of orbital to effective spin moment.

**Table 3:** Room-temperature element-specific magnetic moments of Fe and Co cations (*µ*_0_*H* = 6 T, *T* = 300 K) in 5.1 nm small CFO nanoparticles, bare (S) and Pd decorated (S-Pd), and large 17.8 nm CFO nanoparticles, bare (L) and Pd decorated (L-Pd).

Sample	Fe magnetic moments				Co magnetic moment				Magnetic moment CoFe_2_ (µ_B_)
	*m*_*S*_^*eff*^ (µ_B_)	*m*_*L*_ (µ_B_)	*m*_*L*_*/m*_*S*_^*eff*^ (%)	*m*_*tot*_ (µ_B_)	*m*_*S*_^*eff*^ (µ_B_)	*m*_*L*_ (µ_B_)	*m*_*L*_*/m*_*S*_^*eff*^ (%)	*m*_*tot*_ (µ_B_)	
S	0.32	0.04	11	0.36	0.12	0.02	15	0.14	0.86
S-Pd	0.80	0.00	0	0.80	0.76	0.23	30	0.99	2.59
L	0.47	0.01	2	0.48	0.24	0.07	30	0.31	1.27
L-Pd	0.76	0.00	0	0.76	0.36	0.16	44	0.52	2.04

Uncertainties are 20% for the effective spin moments, 10% for the orbital moment, and 10% for the ratio of orbital to effective spin moment.

At low temperatures of about 5 K, the orbital moments of Fe ions in all samples are zero or very close to zero as expected for Fe^3+^ with its half-filled d shell (3d^5^). In this case, the effective spin moment is basically caused by the spin moment without a significant contribution of the intra-atomic spin dipole term < T_z_ > , which can be related to aspherical spin density distributions. For the case of the small CFO nanoparticles, the spin moment per Fe ion increases from (0.36 ± 0.07) µ_B_ for the bare nanoparticles to (1.1 ± 0.2) µ_B_ for the Pd decorated ones. For the case of large CFO nanoparticles, the relative increase is less pronounced but still clearly detectable, i.e. from (0.76 ± 0.15) µ_B_ to (0.87 ± 0.17) µ_B_. A similar trend is observed for the effective spin moments of Co ions: for the small CFO nanoparticles it increases from about (1.2 ± 0.2) µ_B_ to (1.5 ± 0.3) µ_B_ upon decoration with Pd. For the larger CFO nanoparticles this increase is from (0.81 ± 0.16) µ_B_ to (1.1 ± 0.2) µ_B_. Note that for the case of Co ions a sizeable contribution of < T_z_ > may be present that does not average out due to a larger spin–orbit coupling. The ratios of orbital to effective spin moments of Co are between 41 and 48% for all samples. It seems to be slightly reduced for the Pd decorated samples while the absolute values of orbital moments are larger than for bare CFO. In a single ion model, the large magnetocrystalline anisotropy of CFO was explained by the incomplete quenching of the orbital moment of Co^2+^ ions on octahedral sites^32^, coupling the spin to the axis of trigonal symmetry.

At 300 K, all magnetic moments are reduced with respect to the corresponding values at low temperatures. Again, the total magnetic moments are larger for the Pd decorated CFO nanoparticles. Interestingly, the ratio of orbital to effective spin moment for the Co ions is now significantly enhanced due to the Pd decoration: for the small CFO nanoparticles it increases from 15% to 30% and for the large CFO nanoparticles from 30% to 44%. This suggests a less distinct temperature dependent reduction of the magnetocrystalline anisotropy for Pd decorated nanoparticles. Another feature is the sizeable orbital contribution of Fe for the bare CFO nanoparticles: the small ones exhibit a ratio of orbital to effective spin moment in the order of 10%.

### Element-specific magnetic field-dependent magnetization

In addition, magnetic field-dependent XMCD measurements were performed to investigate the influence of Pd decoration on spin canting effects and on the low field susceptibility relevant for magnetic hyperthermia applications. The field-dependent XMCD was measured at the photon energies of maximum dichroism attributed to Co or Fe ions on octahedral sites. In order to remove non-magnetic artifacts, the data were normalized to field-dependent measurements at photon energies in the pre-edge regions. Around zero magnetic field the vanishing Lorentz force causes a large anomaly in the detected total electron yield (TEY) which is not removed completely by this procedure. Due to the small XMCD—in particular for the small bare CFO nanoparticles—the field-dependent XMCD data were symmetrized, i.e. data of the first and third quadrant were averaged as well as data of the second and fourth quadrant. In several magnetic fields, full spectra were taken and analyzed in addition.

Furthermore, field-dependent XMCD was measured at two different angles of the magnetic field, i.e. perpendicular to the sample plane (0°) and close to in-plane (85°) as shown in Supplementary Figure S8 exemplarily for the large CFO-Pd heterodimers measured at the Co L_3_ edge at 8 K. No significant changes in the coercivity, remanence magnetization or high-field slope were obtained which shows that a possible influence of interparticle interactions like the long-range magnetic dipolar coupling is negligible.

**Figure 6:**
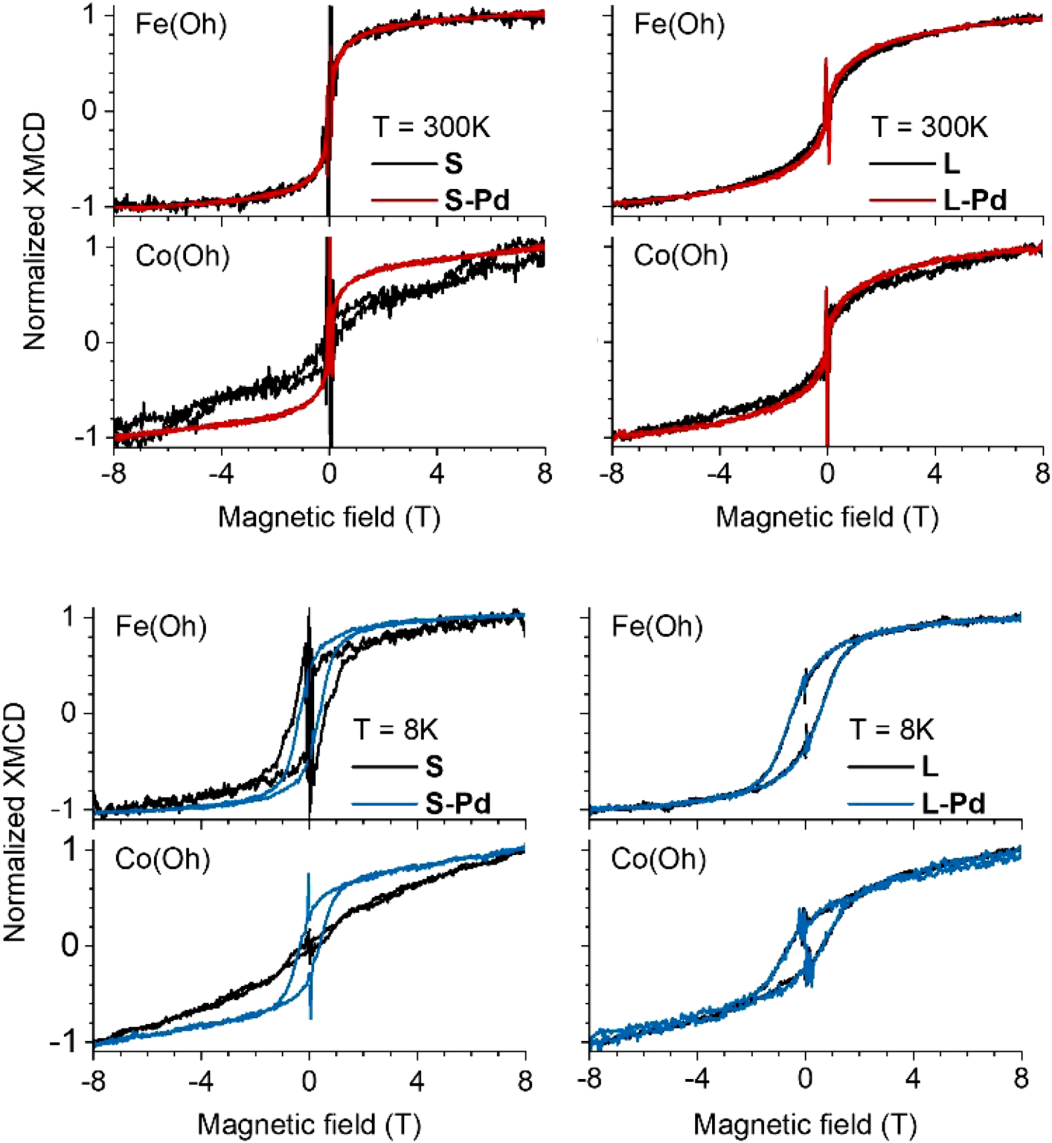
Element-specific field-dependent magnetization. Normalized element-specific field-dependent magnetization for 5.1 nm small CFO nanoparticles, bare (S) and Pd decorated (S-Pd), and large 17.8 nm CFO nanoparticles, bare (L) and Pd decorated (L-Pd) at *T* = 300 K (upper panels) and T = 8 K (lower panels).

The normalized data for both 300 K and 8 K measured at the Co and Fe L_3_ edges are presented in Fig. 6. We start with the results for *T* = 300 K (upper panels). For the small CFO nanoparticles, the field dependence of the magnetic moments of Fe ions on octahedral sites is the same for bare and Pd decorated samples, whereas an obvious change is found for the Co ions. The latter shows very strong spin canting effects in the bare CFO nanoparticles and a similar shape like the Fe ions after Pd decoration. In the region of low magnetic fields, the field dependence of the Co magnetic moment is much steeper after Pd decoration which gives already an explanation for the enhanced maximum heating power in hyperthermia experiments^8^. This trend is also visible for the large CFO nanoparticles, but the effect is much weaker. This size-dependent influence of Pd decoration on the spin canting points towards a distinguished role of the surface of CFO nanoparticles.

To gain further insight into the mechanisms behind the different field-dependent behavior for bare and Pd decorated CFO, field-dependent XMCD was also measured at the low temperature of *T* = 8 K (Fig. 6, lower panels). For the small CFO particles, the spin canting of Co is again reduced upon Pd decoration, while for the large CFO particles no difference between bare and Pd-decorated ones is visible. But in contrast to the data obtained at 300 K, the high-field slopes of Co and Fe ions are different.

This temperature-dependent canting behavior may indicate a significant contribution of magnetic anisotropy. However, a varying degree of electronic localization with temperature, as obtained from XANES analysis, will modify the exchange interactions as well. These different effects are discussed below together with density functional theory results. Furthermore, the influence of Pd which significantly lowers the spin canting of Co ions is also discussed.

### Influence of Pd on competing superexchange interactions

To model the influence of increasing electronic localization, the Fock exchange was increased in hybrid DFT calculations from 20% to 40% and 70%. As expected, the absolute values of magnetic moments increase for all three cations (Supplementary Table S2). In contrast, the dependence of the exchange on the localization can be more complex particularly for the exchange between different ions on *O*_*h*_ sites as has been shown for the case of NiFe_2_O_4_^33^. Since quantification of exchange coupling constants from total energies of different spin configurations is rather error-prone, we restrict this part to a qualitative analysis of hybridization effects by visualizing the uncompensated spin densities to substantiate our conclusions from experimental results.

**Figure 7:**
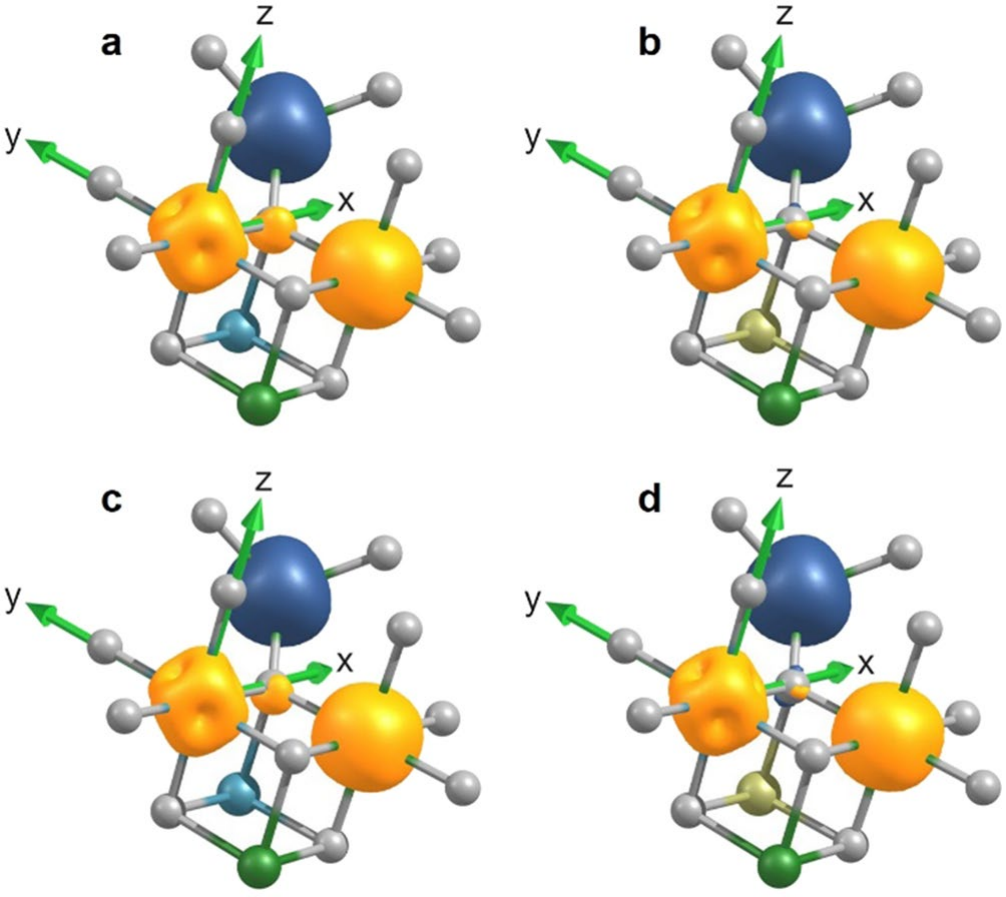
Calculated spin density isosurfaces of CFO and CFO-Pd models. Isosurfaces of spin densities calculated for a 55 atoms cell of CFO. Yellow and blue isosurfaces correspond to majority spin-up and spin-down, respectively. Atom color code: Co—blue, Fe—green, O—grey, Pd—yellow-green. In (**b**,**d**), one Co(O_h_) was replaced by Pd. For clarity, only a few atoms are plotted and the spin densities are shown for one Co(O_h_) ion (located in the origin of the coordinate system), one Fe(O_h_), one Fe(T_d_) and one oxygen anion connecting them. For (**a**,**b**), 40% Fock exchange was used and 70% Fock exchange was used for (**c**,**d**).

In Fig. 7a, the calculated spin density isosurface corresponding to 0.02/r_B_^3^ (r_B_ is the Bohr radius) is shown for CFO using a Fock exchange of 40%. For clarity, only a few atoms of the 55 atom cell and the isosurfaces of only one Co(*O*_*h*_) ion in the origin of the coordinate system, one Fe(*O*_*h*_) ion, one Fe(*T*_*d*_), and the oxygen anion connecting them are depicted. The spin density of Co(*O*_*h*_) resembles the cubic anisotropy, while the almost spherical spin density of Fe(*O*_*h*_) indicates a low anisotropy. The parallel alignment of uncompensated spins of the ions at *O*_*h*_ sites is caused by the dominating intersublattice *O*_*h*_–*T*_*d*_ superexchange interaction. Hybridization with the oxygen anion mediating the superexchange interaction is visible by its spin polarization. Due to the half-filled (Fe^3+^, 3d^5^) or more than half-filled (Co^2+^, 3d^7^) d shell of the transition metal ions, only 2p electrons of the neighboring oxygen anion with minority spin with respect to the 3d cation can hop into the overlapping 3d orbital. Accordingly, the spin polarization of the electrons remaining at the oxygen site in the specific orbital pointing towards the 3d cation has the same sign than the spin polarization of the related 3d cation.

In Fig. 7b, the Co(*O*_*h*_) atom below this oxygen anion was replaced by Pd. Due to the strong crystal field and the delocalized nature of 4d electrons, Pd is in a low-spin (S = 0) state. The delocalized electrons have also a severe impact on the electron density of the neighboring oxygen anions: for the oxygen anion, closer inspected here, we find a smaller electron density compared to CFO in the *p*_*z*_ orbital pointing towards the Pd atom and larger electron densities in both *p*_*x*_ and *p*_*y*_ orbitals. In the coordinate system chosen here, this corresponds to a weakening of the Co(*O*_*h*_)–Fe(*O*_*h*_) superexchange in which the *p*_*x*_ and *p*_*y*_ orbitals are involved. This effect is visible in the smaller spin polarization of the oxygen anion, particularly in the part pointing towards Co(*O*_*h*_). For the same reason, the superexchange is also weakened for the second Co(*O*_*h*_)–Fe(*O*_*h*_) pair in the spinel cube shown in the figure. If we use another CFO configuration and exchange one Fe(*O*_*h*_) with Co(*O*_*h*_) in this part, a weakening of Co(*O*_*h*_)–Co(*O*_*h*_) superexchange is obtained accordingly. In total, this yields a significant modification of the superexchange between ions on *O*_*h*_ sites: for two out of the six neighboring *O*_*h*_ sites the superexchange is weakened, for one it is modified by replacing an *O*_*h*_ cation with Pd. In contrast, the intersublattice superexchange between *O*_*h*_ and *T*_*d*_ sites is much less influenced since it involves also other oxygen orbitals (*p*_*z*_ in the depicted example). As a consequence, the superexchange between *O*_*h*_ and *T*_*d*_ is clearly dominating not only for Fe(*O*_*h*_) as before, but now also for Co(*O*_*h*_) in close vicinity of Pd, thus reducing the spin canting caused by competing exchange interactions in agreement to experimental observations. In this regard, Pd allows the nanoscale CFO to show more bulk-like properties by recovering the intersublattice superexchange as the dominating magnetic coupling.

Interestingly, the spin density isosurface of Co(*O*_*h*_) appears slightly rotated and minima and maxima look more pronounced after replacing one neighboring cation by Pd. A closer inspection shows in fact that the symmetry of the electron density is distorted which indicates that also the magnetocrystalline anisotropy is influenced. Particularly for the small CFO particles, the change in magnetic anisotropy can have a severe impact since it can change the fraction of superparamagnetic particles of the ensemble with certain volume, shape and anisotropy distributions.

In this example we investigated the changes for the case of a Co(*O*_*h*_) ion in a bulk-like surrounding. At the surface in close vicinity to metallic Pd the effects should be even more pronounced, which is in agreement to the experimental result that the influence of Pd is even more significant for the small CFO particles with a larger surface fraction.

At low temperatures, the XANES indicates a stronger electronic localization which has been modeled by a larger contribution of the Fock exchange. In Fig. 7c,d, again the isosurfaces of the spin densities for selected atoms are shown for CFO and CFO-Pd, respectively, including 70% Fock exchange. This localization leads to smaller spin polarization of oxygen anions, connected to weaker superexchange interactions between all neighboring cations. Again, introducing Pd leads to further weakening of Co(*O*_*h*_)–Fe(*O*_*h*_) superexchange in the neighborhood.

**Figure 8:**
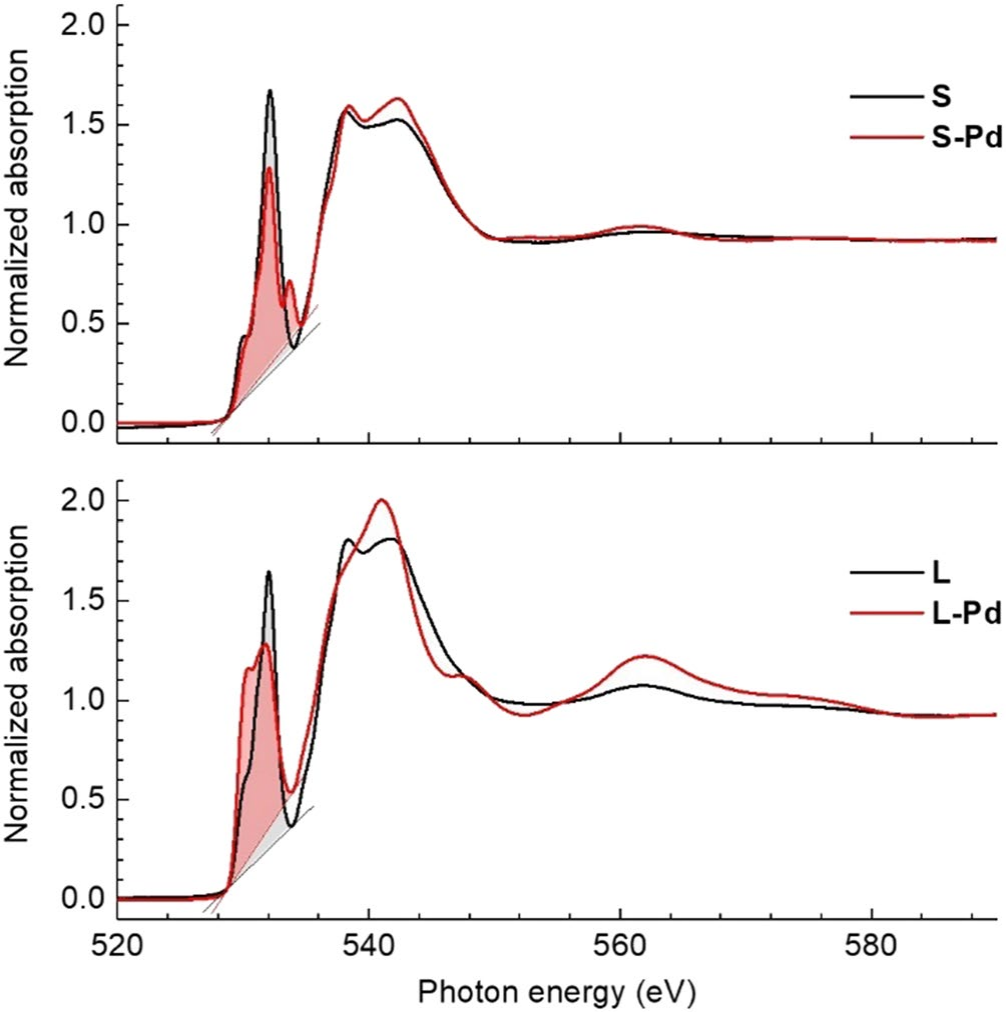
Oxygen K edge spectra. XANES spectra at a temperature of T = 300 K at the *K* absorption edge of oxygen in 5.1 nm small CFO nanoparticles, bare (S) and Pd decorated (S-Pd), top panel, and large 17.8 nm CFO nanoparticles, bare (L) and Pd decorated (L-Pd), bottom panel. The shaded areas mainly correspond to electron transitions into oxygen 2p states hybridized with 3d states. Note that the energy range shown here includes a small contribution (< 2%) of Pd M_3,2_ absorption edges as well.

While the electronic localization can be monitored by the XANES measured at the *L*_3,2_ absorption edges of the 3d transition metal ions, where especially Co exhibits significant changes in its fine structure, a change in the hybridization with oxygen responsible for the modified superexchange, cannot be resolved. A qualitative experimental indication, however, can be found at the oxygen K edge. In Fig. 8, we present the x-ray absorption spectra at the O K edge of small and large CFO nanoparticles with and without Pd decoration at the application-relevant temperature of *T* = 300 K. Although in the measured energy range, also contributions of Pd M_3,2_ absorption edges are expected, the small amount of Pd still allows a qualitative discussion of the oxygen fine structure which changes for both, large and small, CFO after Pd decoration. The main absorption peaks in the energy range 535–554 eV correspond to oxygen 2p states hybridized with 4p, 4s states of the transition metal cations. More relevant in the present context are the absorption peaks in the pre-edge region around 530–532 eV which correspond to electron transitions into oxygen 2p states hybridized with 3d states^34^. The integrated peak intensity (shaded areas) gives an approximation of the hybridization strength^35^. It remains largely unchanged for the large CFO nanoparticles upon decoration with Pd (2.9 in arb. units for the spectra normalized to the energy region far above the edge). In contrast, for the small CFO nanoparticles, Pd decoration leads to a significant decrease of the intensity from 2.7 arb. units for the bare nanoparticles to 2.1 arb. units for CFO-Pd, which qualitatively supports our findings of reduced Co(O_h_)–Fe(O_h_) and Co(O_h_)–Co(O_h_) superexchange via oxygen.

### Influence of magnetic susceptibility on magnetic heating

In order to relate static (element-specific) magnetic properties as investigated in this work to the measured specific absorption rates in AC magnetic fields, the heat generation ability of nanoparticles shall be briefly discussed. The specific absorption rate (*SAR*) is given by the dissipated power *P* per mass of the magnetic material (here, CFO). For magnetic heating by superparamagnetic nanoparticles in an AC magnetic field with small field amplitudes, the dissipated power is given by^36^:

4 $$SAR \propto P=\frac{1}{2}{\mu }_{0}{\chi }_{0}{H}^{2}\omega \frac{\omega \tau }{1+{\left(\omega \tau \right)}^{2}}$$

where ${\mu }_{0}$ is the magnetic constant, ${\chi }_{0}$ is the (static) magnetic susceptibility, $H$ and $\omega$ are amplitude and angular frequency of the external AC magnetic field, respectively, and $\tau$ is the relaxation time. The susceptibility is defined as the derivative of the magnetization with respect to the external magnetic field. This relation shows that in addition to extrinsic parameters of the magnetic field (amplitude and frequency) which are limited by the avoidance of unwanted physiological responses, intrinsic parameters like magnetic susceptibility and relaxation time are important properties to maximize the heating ability.

The influence of magnetic anisotropy, size, and shape that enters the effective relaxation times τ of CFO nanoparticles and CFO-Pd heterodimers have been already discussed in^8^ and cannot be the reason for the significantly enhanced SAR when comparing bare and Pd decorated CFO nanoparticles of the same size. From the experimental results obtained in this work, the increase in static magnetic susceptibility at 300 K becomes obvious: the combination of larger element-specific net magnetic moments and reduced spin canting, particularly for the Co ions, lead to the higher magnetic susceptibility in the application-relevant small magnetic fields up to about 1 T.

## Summary and conclusions

Hybrid nanostructures of CFO-Pd heterodimers were successfully synthesized in two ranges of particle size using a two-pot modified coprecipitation method. Microscopic analyses demonstrate the synthesis of magnetic cobalt ferrite nanoparticles of significant crystallinity and with homogeneous shape and size distribution, as well as the random decoration of their surfaces with ultrasmall metallic palladium nanoparticles^19^. The decoration with Pd leads to (1) increased effective spin and orbital moments for both, Fe and Co, and (2) less spin canting effects for Co ions.

The effects are remarkably strong, more significant for the small CFO nanoparticles and physically explain the higher magnetic heating power of Pd decorated nanoparticles^8^. In particular, the large changes in the field-dependent magnetization point towards a key role of Co ions in this regard.

From DFT calculations, a plausible explanation for this enhanced sensitivity of Co ions is the weakening of Co(O_h_)–Fe(O_h_) and Co(O_h_)–Co(O_h_) superexchange in close vicinity to Pd atoms that, in comparison, reinforce the superexchange between O_h_ and T_d_ sites as the dominating one for all cations. The complex interplay between different exchange mechanisms connected to electronic (de-)localization, possible shielding effects as well as modifications of magnetic anisotropy when combining ferrites with atoms exhibiting a more delocalized electronic structure may further stimulate theoretical investigations of such heterostructures.

Achievements of this work contribute to a more comprehensive understanding of the magnetic interactions and properties in hybrid nanoparticles and also elucidate a promising path for the development of other metal-ferrite nanohybrids.

## Methods

### Synthesis and preparation

CFO-Pd hybrid nanoparticles were fabricated via two successive steps: in the first step, the synthesis of superparamagnetic CFO nanoparticles was carried out using a modified coprecipitation method using NaOH solution and chelating agents which have been explained in our previous publications^23,24^. For the formation of CFO-Pd heterodimers in the second step, the surfaces of synthesized CFO nanoparticles were initially activated and then the metallic palladium nanoclusters were precipitated from an aqueous solution of K_2_PdCl_4_ intermediate phase. More details of the synthesis process of CFO and CFO-Pd nanoparticles are presented in the Supplementary Information. Supplementary Scheme S1 demonstrates the stepwise synthesis process of CFO-Pd nano-heterodimers.

The bare CFO nanoparticles have already been characterized regarding their size and morphology as well as cation disorder and averaged magnetic properties by various methods, such as X-ray diffraction and Rietveld refinement analysis^37,38^, dynamic light scattering^37^ (high-resolution) transmission electron microscopy ((HR-)TEM) including energy-dispersive X-ray spectroscopy (EDS)^8,23,24^ and vibrating sample magnetometry (VSM) at 300 K^8,23,24^. Elemental studies on the Pd decorated CFO-Pd heterodimers were carried out by use of ICP-OES. For X-ray absorption studies, the samples were prepared by drop casting of the ferrofluid on polished p-doped silicon wafers and drying in vacuum at ambient temperature.

### Magnetometry

Magnetometry data at 300 K using the same samples and instrument have already been published in^8^. Additional magnetometry measurements at low temperature of T = 8 K were carried out on powder samples using the VSM (VSM-PPMS-14T). For the measurements, the powder samples were pressed in a piston-cylinder fashion using standard polypropylene capsules. The weights of the samples were determined using a microbalance for the empty capsules and the filled ones.

### Atomic force microscopy

AFM was used to image the morphology of the drop-casted samples. The AFM images were recorded in tapping™ mode using a Cypher™ Scanning Probe Microscope (Asylum Research, Oxford Instruments). All images were recorded at room temperature in air with 512 × 512 pixel resolution using a scan rate of 2.44 Hz. Images were recorded in the repulsive mode. For this paper, in order to enhance contrast and visibility, the AFM images were leveled using a home-written MATLAB ^®^ 2014a code comprising a line-by-line leveling based on a polynomial leveling of second order and a final histogram spread to maximize contrast.

### X-ray absorption spectroscopy

High-resolution X-ray absorption spectra were measured in the high-field end station at the undulator beamline UE46-PGM1^39^ at the synchrotron radiation source BESSY II of the Helmholtz-Zentrum Berlin (HZB). XMCD spectra were taken in magnetic fields of 6 T applied (anti-) parallel to the k vector of incident X-rays with either positive or negative helicity. The degree of circular polarization was 90%. To record X-ray magnetic linear dichroism (XMLD) the magnet was rotated by 90° giving the opportunity to measure absorption with linearly polarized X-rays with their electric field vector parallel (horizontal polarization) or perpendicular (vertical polarization) to the magnetic field axis. Measurements were performed at the Fe L_3,2_ absorption edges (680 eV–780 eV) and Co L_3,2_ absorption edges (770 eV–840 eV) at *T* = 5 K and 300 K, respectively, in the surface sensitive TEY mode.

Magnetic-field dependent element-specific magnetization curves were measured in the VEKMAG end station at the bending magnet beamline PM2^40^ at the HZB-BESSY II synchrotron radiation source in magnetic fields between ± 8 T with fast ramp rates of 2 T/min. The degree of circular polarization was about 77%.

### Atomic multiplet calculations

Charge transfer multiplet calculations were performed to analyze the spectral shape of experimental X-ray absorption spectra. For this purpose, the CTM4XAS program package^41^ has been used. It is based on a semi-empirical approach including crystal field theory, core–hole effects, spin–orbit coupling, multiplet effects as well as charge transfer.

For the case of Fe^3+^ ions on octahedral sites, we used a crystal field splitting of 10Dq =  + 1.8 eV, and reduced values of the Slater integral of 80% of the atomic value accounting for a reduced Coulomb repulsion. For Fe^3+^ ions on tetrahedral sites a negative crystal field splitting of 10Dq = − 0.8 eV was used. The negative sign considers the reversed order of t_2_ and e orbital energies with respect to the octahedral case. The reduced absolute value reflects the different spatial distribution of orbitals with respect to the positions of negatively charged oxygen anions creating the crystal field.

For the case of Co^2+^ ions, the crystal fields are smaller because of the lower charge of the cation. Here, we used 10Dq =  + 1.1 eV and 10Dq = − 0.6 eV for the octahedral and tetrahedral case, respectively. The values of the Slater integrals were reduced to 90% of the atomic value. For all other quantities, corresponding atomic values have been used. Lorentzian life-time broadenings of 0.2 eV and 0.5 eV for the L_3_ and L_2_ absorption edge, respectively, were included. An additional Gaussian broadening of 0.1 eV considers the instrumental broadening.

### Density functional theory

Hybrid Hartree–Fock density functional theory calculations were performed with Perdew–Burke–Ernzerhof (PBE)^42^ and Fock exchange and PBE correlation functional according to:

5 $$E_{{{\text{XC}}}} = E_{{\text{X}}}^{{{\text{PBE}}}} + a\left( {E_{{\text{X}}}^{{\text{F}}} - E_{{\text{X}}}^{{{\text{PBE}}}} } \right) + E_{{\text{C}}}^{{{\text{PBE}}}}$$

where *E*_X_^PBE^, *E*_C_^PBE^, and *E*_X_^F^ denote the exchange and correlation parts of the PBE density functional and the Fock exchange functional, respectively^43^. The local part of the correlation functional was parametrized due to Perdew and Wang (PW91)^44^. The def2-TZVP^45^ and def2/J^46^ basis sets were used as implemented in the ORCA package^47^. To reduce computation time, the auxiliary RIJCOSX basis set was employed^48^. In order to investigate the influence of the degree of localization on the electronic and magnetic properties, which is an important issue for ferrites, different mixtures of DFT and exact Hartree–Fock (HF) exchange functionals were used, i.e. a fraction of 20%, 40% as suggested for NiFe_2_O_4_^33^, and 70% Fock exchange. A small cluster of CFO with lattice constant *a* = 0.838 nm with a diameter of about one unit cell was used as structural input. In more detail, three Co ions at O_h_ sites, five Fe ions at O_h_ sites and six Fe ions at T_d_ sites were included as well as all 41 oxygen ions necessary to define their coordination symmetry. After calculating the artificial high-spin state of CFO, a broken symmetry calculation was performed with reversed spins of Fe ions at T_d_ sites. In the next step, one Co ion was replaced by Pd, for which an effective core potential (ECP)^49^ was introduced. To find self-consistent field (SCF) solutions, default values for convergence tolerances were used, i.e. for the energy change 1 × 10^−6^*E*_h,_ where *E*_h_ denotes the Hartree energy, the maximum density change was limited to 1 × 10^−5^, average (root mean square) density change: 1 × 10^−6^, and the error in direct inversion in iterative subspace (DIIS): 1 × 10^−6^ *r*_B_ where *r*_*B*_ is the Bohr radius. To achieve convergence, the maximum number of Fock matrices to remember was increased to 15 and in the beginning of the SCF iterations additional damping was introduced. For visualization of spin densities, the multiwfm software, version 3.6, was used^50^.

## Supplementary Information

Supplementary Information 1.

## Data Availability

The datasets generated and/or analyzed during the current study are available from the corresponding author on reasonable request.

## References

[1] Ghazanfari MR, Kashefi M, Shams SF, Jaafari MR (2016). Perspective of Fe_3_O_4_ nanoparticles role in biomedical applications. Biochem. Res. Int..

[2] Bogart LK, Pourroy G, Murphy CJ, Puntes V, Pellegrino T, Rosenblum D, Peer D, Lévy R (2014). Nanoparticles for imaging, sensing, and therapeutic intervention. ACS Nano.

[3] Seeta Rama Raju G, Benton L, Pavitra E, Yu JS (2015). Multifunctional nanoparticles: Recent progress in cancer therapeutics. Chem. Commun..

[4] Kalidindi SB, Jagirdar BR (2012). Nanocatalysis and prospects of green chemistry. Chemsuschem.

[5] Xie J, Liu G, Eden HS, Ai H, Chen X (2011). Surface-engineered magnetic nanoparticle platforms for cancer imaging and therapy. Acc. Chem. Res..

[6] Gallo J, Long NJ, Aboagye EO (2013). Magnetic nanoparticles as contrast agents in the diagnosis and treatment of cancer. Chem. Soc. Rev..

[7] Pan Y, Du XW, Zhao F, Xu B (2012). Magnetic nanoparticles for the manipulation of proteins and cells. Chem. Soc. Rev..

[8] Shams SF, Ghazanfari MR, Pettinger S, Tavabi AH, Siemensmeyer K, Smekhova A, Dunin-Borkowski RE, Westmeyer GG, Schmitz-Antoniak C (2020). Improved specific absorption rates in CoFe_2_O_4_ nanohybrids: A magnetc fluid hyperthermia perspective, 2019. Phys. Chem. Chem. Phys..

[9] Maaz K, Mumtaz A, Hasanain SK, Ceylan A (2007). Synthesis and magnetic properties of cobalt ferrite (CoFe_2_O_4_) nanoparticles prepared by wet chemical route. J. Magnet. Magnet. Mat..

[10] Shafi KVPM, Gedanken A, Prozorov R, Balogh J (1998). Sonochemical preparation and size-dependent properties of nanostructured CoFe_2_O_4_ particles. Chem. Mater..

[11] Wang BY, Singh SB, Shao YC, Wang YF, Chuang CH, Yeh PH, Chiou JW, Pao CW, Tsai HM, Lin HJ, Lee JF, Tsai CY, Hsieh WF, Tsai M-H, Pong WF (2013). Effect of geometry on the magnetic properties of CoFe_2_O_4_–PbTiO_3_ multiferroic composites. RSC Adv..

[12] Carta D, Casula MF, Falqui A, Loche D, Mountjoy G, Sangregorio C, Corrias A (2009). A structural and magnetic investigation of the inversion degree in ferrite nanocrystals MFe_2_O_4_ (M = Mn Co, Ni). J. Phys. Chem. C.

[13] Sawatzky GA, van der Woude F, Morrish AH (1968). Cation distributions in octahedral and tetrahedral sites of the ferrimagnetic spinel CoFe_2_O_4_. J. Appl. Phys..

[14] Margulies DT, Parker FT, Rudee ML, Spada FE, Chapman JN, Aitchison PR, Berkowitz AE (1997). Origin of the anomalous magnetic-behavior in single-crystal Fe_3_O_4_ films. Phys. Rev. Lett..

[15] Peddis D, Cannas C, Piccaluga G, Agostinelli E, Fiorani D (2010). Spin-glass-like freezing and enhanced magnetization in ultra-small CoFe_2_O_4_ nanoparticles. Nanotechnology.

[16] Peddis D, Yaacoub N, Ferretti M, Martinelli A, Piccaluga G, Musinu A, Cannas C, Navarra G, Greneche JM, Fiorani D (2011). Cationic distribution and spin canting in CoFe_2_O_4_ nanoparticles. J. Phys. Condens. Matter.

[17] Ansari SM, Kashid V, Salunke H, Sen D, Kolekar YD, Ramana CV (2020). First-principles calculations of the electronic structure and magnetism of nanostructured CoFe_2_O_4_ microgranules and nanoparticles. Phys. Rev. B.

[18] Carta D, Mountjoy G, Navarra G, Casula MF, Loche D, Marras S, Corrias A (2007). X-ray absorption investigation of the formation of cobalt ferrite nanoparticles in an aerogel silica matrix. J. Phys. Chem. C.

[19] Vaingankar AS, Patil SA, Sahasrabudhe VS (1980). Degree of inversion in cobalt ferrite by EXAFS (Conference Paper). Trans. Ind. Inst. Met..

[20] Liu J, Römer I, Tang SVY, Valsami-Jones E, Palmer RE (2017). Crystallinity depends on choice of iron salt precursor in the continuous hydrothermal synthesis of Fe–Co oxide nanoparticles. RSC Adv..

[21] Venkatesan K, Babu DR, Bai MPK, Supriya R, Vidya R, Madeswaran S, Anandan P, Arivanandhan M, Hayakawa Y (2015). Structural and magnetic properties of cobalt-doped iron oxide nanoparticles prepared by solution combustion method for biomedical applications. Int. J. Nanomed..

[22] Soler MAG, Lima ECD, da Silva SW, Melo TFO, Pimenta ACM, Sinnecker JP, Azevedo RB, Garg VK, Oliveira AC, Novak MA, Morais PC (2007). Aging investigation of cobalt ferrite nanoparticles in low pH magnetic fluid. Langmuir.

[23] Shams SF, Kashefi M, Schmitz-Antoniak C (2017). Statistical approach of synthesize CoFe2O4 nanoparticles to optimize their characteristics using response surface methodology. J. Magn. Magn. Mater..

[24] Shams SF, Kashefi M, Schmitz-Antoniak C (2018). Rietveld structure refinement to optimize the correlation between cation disordering and magnetic features of CoFe2O4 nanoparticles. New J. Chem..

[25] Bhat PB, Inam F, Bhat BR (2014). Nickel hydroxide/cobalt–ferrite magnetic nanocatalyst for alcohol oxidation. ACS Combin. Sci..

[26] Goroncy C, Saloga PEJ, Gruner M, Schmudde M, Vonnemann J, Otero E, Haag R, Graf C (2018). Influence of organic ligands on the surface oxidation state and magnetic properties of iron oxide particles. Z. Phys. Chem..

[27] Hasz K, Ijiri Y (2014). Particle moment canting in CoFe_2_O_4_ nanoparticles. Phys. Rev. B.

[28] van der Laan G, Arenholz E, Chopdekar RV, Suzuki Y (2008). Influence of crystal field on anisotropic X-ray magnetic linear dichroism at the Co^2+^ L_2,3_ edges. Phys. Rev. B.

[29] Thole BT, Carra P, Sette F, van der Laan G (1992). X-ray circular dichroism as a probe of orbital magnetization. Phys. Rev. Lett..

[30] Carra P, Thole BT, Altarelli M, Wang X (1993). X-ray circular dichroism and local magnetic fields. Phys. Rev. Lett..

[31] Chen CT, Idzerda YU, Lin H, Smith NV, Meigs G, Chaban E, Ho GH, Pellegrin E, Sette F (1995). Experimental confirmation of the x-ray magnetic circular dichroism sum rules for iron and cobalt. Phys. Rev. Lett..

[32] Slonczewski JC (1958). Origin of magnetic anisotropy in cobalt-substituted magnetite. Phys. Rev..

[33] Andrae D, Häußermann U, Dolg M, Stoll H, Preuß H (1990). Energy-adjusted ab initio pseudopotentials for the second and third row transition elements. Theor. Chim. Acta.

[34] de Groot FMF, Gnom M, Fuggle JC, Ghijsen J, Sawatzky GA, Petersen H (1989). Oxygen 1s X-ray-absorption edges of transition-metal oxides. Phys. Rev. B.

[35] Suntivich J, Hong WT, Lee Y-L, Rondinelli JM, Yang W, Goodenough JB, Dabrowski B, Freeland JW, Shao-Horn Y (2014). Estimating hybridization of transition metal and oxygen states in perovskites from O K-edge X-ray absorption spectroscopy. J. Phys. Chem. C.

[36] Rosensweig RE (2002). Heating magnetic fluid with alternating magnetic field. J. Magnet. Manget. Mater..

[37] Shubitidze F, Kekalo K, Stigliano R, Baker I (2015). Magnetic nanoparticles with high specific absorption rate of electromagnetic energy at low field strength for hyperthermia therapy. J. App. Phys..

[38] Torres TE, Lima E, Mayoral A, Ibarra A, Marquina C, Ibarra MR, Goya GF (2015). Validity of the Neel–Arrhenius model for highly anisotropic CoxFe3-xO4 nanoparticles. J. App. Phys..

[39] Helmholtz-Zentrum Berlin für Materialien und Energie (2018). The UE46 PGM-1 beamline at BESSY II. J. Large Scale Res. Facil..

[40] Noll T, Radu F (2016). The mechanics of the Vekmag experiment. Proc. MEDSI2016..

[41] Stavistrki E, de Groot FMF (2010). The CTM4XAS program for EELS and XAS spectral shape analysis of transition metal L edges. Micron.

[42] Perdew JP, Burke K, Ernzerhof M (1996). Generalized gradient approximation made simple. Phys. Rev. Lett..

[43] Adamo C, Barone V (1999). Toward reliable density functional methods without adjustable parameters: The PBE0 model. J. Chem. Phys..

[44] Perdew JP, Wang Y (1992). Accurate and simple analytic representation of the electron-gas correlation energy. Phys. Rev. B.

[45] Weigend F, Ahlrichs R (2005). Balanced basis sets of split valence, triple zeta valence and quadruple zeta valence quality for H to Rn: Design and assessment of accuracy. Phys. Chem. Chem. Phys..

[46] Weigend F (2006). Accurate Coulomb-fitting basis sets for H to Rn. Phys. Chem. Chem. Phys..

[47] Neese F (2012). The ORCA program system. Wiley Interdiscip. Rev..

[48] Neese F, Wennmohs F, Hansen A, Becker U (2009). Efficient, approximate and parallel Hartree-Fock and hybrid DFT calculations. A ‘chain-of-spheres’ algorithm for the Hartree-Fock exchange. Chem. Phys..

[49] Zhou X, Yan S, Barbiellini B, Harris VG, Vittoria C (2006). A computational study of nickel ferrite. J. Magn. Magn. Mat..

[50] Lu T, Chen F (2012). Multiwfn: A multifunctional wavefunction analyzer. J. Comput. Chem..

