# Supplementary material for: Element-specific contributions to improved magnetic heating of theranostic CoFe2O4 nanoparticles decorated with Pd
Source: Sci Rep. 2021 Aug 4;11:15843. doi: 10.1038/s41598-021-95189-y (PMC8338997; doi:10.1038/s41598-021-95189-y)
Supplement: Supplementary file 1 — Supplementary Information 1. [file 41598_2021_95189_MOESM1_ESM.pdf]

Supplementary Information

Element-specific contributions to improved magnetic heating of theranostic $CoFe_2O_4$ nanoparticles decorated with Pd

S. Fatemeh Shams,[1,*] Detlef Schmitz,[2] Alevtina Smekhova,[1,#] Mohammad Reza Ghazanfari,[3] Margret Giesen,[1] Eugen Weschke,[2] Kai Chen,[2] Chen Luo,[2] Florin Radu,[2] Carolin Schmitz-Antoniak[1]

1. *Peter-Grünberg-Institut (PGI-6), Forschungszentrum Jülich, 52425 Jülich, Germany*
2. *Helmholtz-Zentrum Berlin für Materialien und Energie, 14109 Berlin, Germany*
3. *Institute of Chemistry and Biochemistry, Freie Universität Berlin, 14195 Berlin, Germany*

# Present address: Helmholtz-Zentrum Berlin für Materialien und Energie, 12489 Berlin, Germany

**: corresponding author, E-Mail: F.Shams@fz-juelich.de*

1. Synthesis and structural characterization of CFO and CFO-Pd nanoparticles

To synthesize CFO nanoparticles, $FeCl_3.6H_2O$ and $CoCl_2.6H_2O$ (>99%, Merck) were used as precursors, NaOH (1 M, Merck) as an adjusting agent of solution pH, and the citric and nitric acids (>99.5%, >65%, Merck) as chelating agents. Moreover, the required materials during decoration process of metallic Pd nanoparticles on the CFO surfaces included $PdCl_2$ (>99%, Merck), KCl (>99.5%), HCl (37-38%), and citric acid. All materials, solutions, and surfactants were used without further purification.

CFO-Pd hybrid nanoparticles were fabricated via two successive steps: In the first step, the synthesis of superparamagnetic CFO nanoparticles was carried out using a modified coprecipitation method. The suitable conditions for precipitation of the ferrite phase were provided by adjusting of medium pH value using NaOH solution (1 M). The critical parameters of synthesis process were selected and controlled according to the so-called design of experiments approach. Moreover, citric and nitric acids were used as chelating agents to control the size, size distribution, and physical stability of nanoparticles which were dispersed in a colloidal ferrofluid after magnetic separation and washing. For the formation of CFO-Pd heterodimers in the second step, the surfaces of CFO nanoparticles were initially activated through addition of 2 vol.% of HCl solution to the pH-neutralized ferrofluid colloid and subsequent sonication for 20 min. As a result, not only the agglomerated structures were separated and dispersed, but also the CFO surfaces were activated via covering by a film consisting of hydroxyl groups to connect ultrasmall Pd nanoparticles. A water-soluble intermediate phase of K_2PdCl_4 was created using a specific mixture of $PdCl_2$ and KCl salts. After 30 min, the CFO nanoparticles were added, homogenized at room temperature for 60 min, followed by adding NaOH solution for pH adjustment in the range of 12-13, and incubation for 24 h under N_2 atmosphere. Finally, the prepared samples were magnetically separated, centrifuged, washed with deionized water and ethanol, pH-neutralized, and dried in a vacuum oven. The hydroxyl groups derived during the reaction led to hydrophilicity of hybrid nanoparticles surfaces.

According to the dynamic light scattering (DLS) histograms, the average particles hydrodynamic diameter equals 7.2 nm for small nanoparticles and 24.10 nm for large ones while their deviation from the mean value (diameter distribution) is corresponding to 2.5 and 8 nm, respectively. However, since DLS analysis reveals the hydrodynamic diameter of nanoparticles, their geometric diameter is slightly smaller with narrower distribution. Moreover, the undersize limits of small and large samples are equal to 9 and 29 nm, respectively. DLS and undersize plots obviously

show the samples in both particles size ranges are appropriate to be employed in biomedical applications without vascular obstruction, identification by immune system, and severe accumulation in organs [S1-S6].

The compositional and structural characteristics as well as size and size distribution of nanoparticles were evaluated via x-ray diffraction using Cu $K\alpha_{1,2}$ radiation at 40 kV and 40 mA at ambient temperature (by a 2-theta range of 20–80° and a step size and rate of 0.041° and 5 seconds, respectively) of an X-ray diffractometer (XRD, Bruker Advance 2). The XRD results of bare CFO samples merely show the peaks of cobalt ferrite crystallized in inverse spinel structure that confirm the absence of any undesired intermediate phases such as cobalt oxide (CoO). The weighted profile factors (R_{wp}) of experimental results of XRD analyses with reference pattern with CIF # 1533163 are less than 10 for both samples which are acceptable values. Considering the wet-chemical synthesis process and lack of micro-strain in particles structure, the comparative peaks broadening remarkably at 2ϴ>50° could be purely ascribed to the nanocrystalline structure of samples.

Qualitatively, there is no notable difference between XRD patterns of the samples. Based on quantitative analyses of Rietveld structure refinement, the crystallite sizes are equal to 5.1 and 17.8 nm for small and large samples, in agreement with the slightly larger hydrodynamic diameters determined by DLS. Furthermore, the crystallinity degrees of $CoFe_2O_4$ phase are about 82% and 85% in small and large samples, respectively. A non-crystalline fraction of synthesized nanoparticles could be attributed to partial creation of an amorphous phase (especially in small nanoparticles), and also negligible amounts of non-target phases in particular in the large nanoparticles. In addition, the Rietveld refinement results prove the calculable cation disorder of Co and Fe ions in both samples which directly affects their magnetic properties. The magnetic cations of small nanoparticles are more disordered from ideal inverse spinel structure compared to the large ones.

2. Mass Magnetometry

At the low temperature of 8 K, all CFO samples are magnetically blocked. Upon decoration with Pd, the values of high-field magnetization and remanent magnetization (M_r) are remarkably enhanced. In contrast, the shells of noble metals in particular diamagnetic elements such as copper, silver, and gold weaken the magnetization of magnetic cores [S7,S8]. Fig. S3 shows the M-H hysteresis loops measured at 8 K and confirms the ferrimagnetic behavior of the particles at 8 K. The negative susceptibility at the beginning of the virgin magnetization curve is common for frustrated ferrimagnetic systems and points towards a spin-glass-like behavior. Even in high external magnetic fields of 10 T and above, magnetic saturation is not reached. In pure CFO samples, the large nanoparticles exhibit higher magnetization in agreement with the magnetic moments determined from XMCD measurements. However, after decorating with Pd clusters the magnetization at high fields of the large particles is similar to the one of the smaller particles. This finding could be explained by the larger surface fraction of the small nanoparticles and the concomitant higher fraction of Pd decoration in comparison with the large nanoparticles.

Following the approach of a magnetically "dead" surface layer, the reduced magnetization can be related to a layer of thickness t_d that does not contribute to the net magnetization according to [S9]

$$t_d \approx \frac{d}{6}\left(1 - \frac{M_S}{M_S^{bulk}}\right) \qquad \text{(S1)}$$

Since we did not reach the saturation magnetization, we insert the magnetization in 13 T in eq. (S1) instead which leads to an estimated upper limit for the "dead" layer thickness. Based on this relation, t_d decreases from 1.8 nm to 1.2 nm (0.7 nm to 0.3 nm) for the large (small) CFO nanoparticles upon decoration with Pd.

The effective magnetic anisotropy comprising of magnetocrystalline anisotropy, shape anisotropy, surface anisotropy, and magnetoelastic anisotropy can be estimated from the coercive fields and magnetization values. According to the Stoner-Wohlfarth model for non-interacting nanoparticles with randomly oriented easy axes of uniaxial effective anisotropy, the effective anisotropy constant is approximated by

$$K_{eff} = \mu_0 H_C M_S \qquad \text{(S2)}$$

Since the saturation magnetization was not reached, we take the values in the highest external magnetic applied here, i.e. in 13 T, which yields a lower limit of the real effective anisotropy.

The calculated values are summarized in **Supplementary Table S1**. The effective anisotropy density constants in J/m^3 were obtained by multiplication with the density $\rho = 5.3 \times 10^3$ kg/m^3 and are in reasonable agreement with the values determined at 300K in ref. [8] in the manuscript with fitting the law-of-approach to saturation to experimental data.

For the sake of completeness, magnetometry data at a temperature of 300 K measured for increasing and decreasing magnetic field directions are shown in **Supplementary Figure S4**. Note that some of these data were published in the Supplementary Information of Ref. [8]. Coercive fields and remanence magnetization vanish within experimental uncertainties as expected for superparamagnetic nanoparticles. The high-field magnetization in 13 T is significantly smaller compared to values obtained at 8 K.

3. X-ray absorption spectroscopy: data treatment and application of sum rules

To obtain the whole sets of XANES, XMCD, and XMLD, measurements were performed in two geometries: For XMCD the magnetic field was applied parallel or antiparallel to the k vector of incoming circularly polarized X-rays. According to the positive or negative helicity, the spectra are denoted I_+ and I_- in the following. Measurements were performed in normal incidence, i.e. the k vector of X-rays was perpendicular to the substrate surface. For XMLD measurements the magnetic field was rotated by 90° and the x-ray polarization was changed to either horizontal (E field parallel to the magnetic field axis, $I_\parallel$) or vertical (E field perpendicular to the magnetic field axis, $I_\perp$). To obtain a reasonable TEY signal in this geometry, the sample was rotated by about 8° off normal incidence.

The spectra were corrected by scaling with a linear function to fit the I_- spectrum in the pre-edge and post-edge regions. For all spectra, the scaling factor was well below 1% of the signal. Related to the different measurement geometries, an additional linear background had to be taken into account for the $I_\parallel$ and $I_\perp$ spectra.

The 3d XANES was obtained by the following procedure:

(i) averaging I_- and the background corrected I_+ and $I_\parallel$ spectra according to

$$(I_- + I_+ + I_\parallel)/3 \qquad \text{(S3)}$$

(ii) subtracting a linear fit to the pre-edge region
(iii) division by a constant, so that averaged spectra match unity in the post-edge region
(iv) subtract a two step-like function to separate electron transitions to unoccupied 3d states from transitions into higher unoccupied states or the continuum.

The integrals of the 3d XANES spectra are proportional to the number of unoccupied 3d states.

We used fixed values of the free ions, i.e. n_h = 5 for Fe^{3+} and n_h = 3 for $Co2^+$, for the large bare CFO nanoparticles. As energy-dependent two step-like function we used two Fermi functions:

$$\frac{2/3}{1+\exp[E(L_3)-E]} + \frac{1/3}{1+\exp[E(L_2)-E]} \qquad \text{(S4)}$$

In this equation $E(L_3)$ denotes the energy position (inflection point) of the L_3 absorption edge and $E(L_2)$denotes the energy position (inflection point) of the L_2 absorption edge. The weighting of the two steps with 2/3 and 1/3 reflects the occupation of the initial $2p_{3/2}$ and $2p_{1/2}$ states, respectively.

For other samples, the numbers of unoccupied final states have been carefully renormalized for the sum rules based analysis. The largest deviation was found for the small bare CFO nanoparticles at T = 5 K, where for Fe n_h = 4.7 was found and for Co n_h = 3.3.

The normalization steps (ii) and (iii) were applied to the initial spectra as well. The XMCD spectra were obtained from

$$(I_{+}^{norm} - I_{-}^{norm})/P \quad \text{(S5)}$$

where P is the degree of circular polarization (in our case $P = 0.9$). The XMLD spectra were calculated according to

$$(I_{\parallel}^{norm} - I_{\perp}^{norm}) \quad \text{(S6)}$$

5. Supplementary tables

Table S1: Low temperature (8 K) coercivity μ_0H_c, remanent magnetization M_r and high-field magnetization M(13T) for bare and Pd decorated samples of different sizes; small (S) and large (L). Estimated values of the low-temperature effective anisotropy constant have been calculated according to eq. (S2) and give lower limits of the real values. For comparison, effective anisotropies at 300K [8] were added.

Sample	μ_0H_c (T)	M_r (Am^2/kg)	M(13T) (Am^2/kg)	K_{eff} (J/kg)	K_{eff} (10^5 J/m^3)	K_{eff} (10^5 J/m^3) at T = 300K [8]
**S**	0.70 ± 0.02	6.7 ± 0.2	18.1 ± 0.3	12.6 ± 0.5	0.7 ± 0.5	0.4 ± 0.1
**S-Pd**	0.73 ± 0.02	26.1 ± 0.2	53.4 ± 0.3	39.0 ± 0.5	2.1 ± 0.5	2.7 ± 0.4
**L**	0.86 ± 0.02	12.4 ± 0.2	34.6 ± 0.3	29.8 ± 0.5	1.6 ± 0.5	1.5 ± 0.3
**L-Pd**	0.88 ± 0.02	22.9 ± 0.2	53.7 ± 0.3	47.3 ± 0.5	2.5 ± 0.5	2.9 ± 0.4

Table S2. Calculated 3d magnetic moments of Co and Fe on octahedral (O_h) or tetrahedral (T_d) lattice sites by DFT for a fixed crystal structure using the bulk lattice constant (8.38 Å). Details of DFT calculations are given in the method section of the main text.

System	Fock exchange	Co(O_h)	Fe(O_h)	Fe(T_d)	Fe(O_h)+Fe(T_d)	Co(O_h)+Fe(O_h)+Fe(T_d)
**CFO**	20%	2.74 μ_B	4.08μ_B	-3.95μ_B	0.13 μ_B	2.87 μ_B
**CFO**	40%	2.75μ_B	4.35 μ_B	-4.19 μ_B	0.16 μ_B	2.91 μ_B
**CFO**	70%	2.83 μ_B	4.66 μ_B	-4.42 μ_B	0.24 μ_B	3.07 μ_B
**CFO-Pd**	20%	2.67 μ_B	4.16 μ_B	-4.03 μ_B	0.13 μ_B	2.80 μ_B
**CFO-Pd**	40%	2.73 μ_B	4.35 μ_B	-4.18 μ_B	0.17 μ_B	2.90 μ_B
**CFO-Pd**	70%	2.82 μ_B	4.54 μ_B	-4.39 μ_B	0.15 μ_B	2.97 μ_B

6. Supplementary figures and schemes

Scheme S1: Graphical illustration of stepwise synthesis process of CFO nanoparticles and Hybrid nanostructures of CFO-Pd heterodimers.

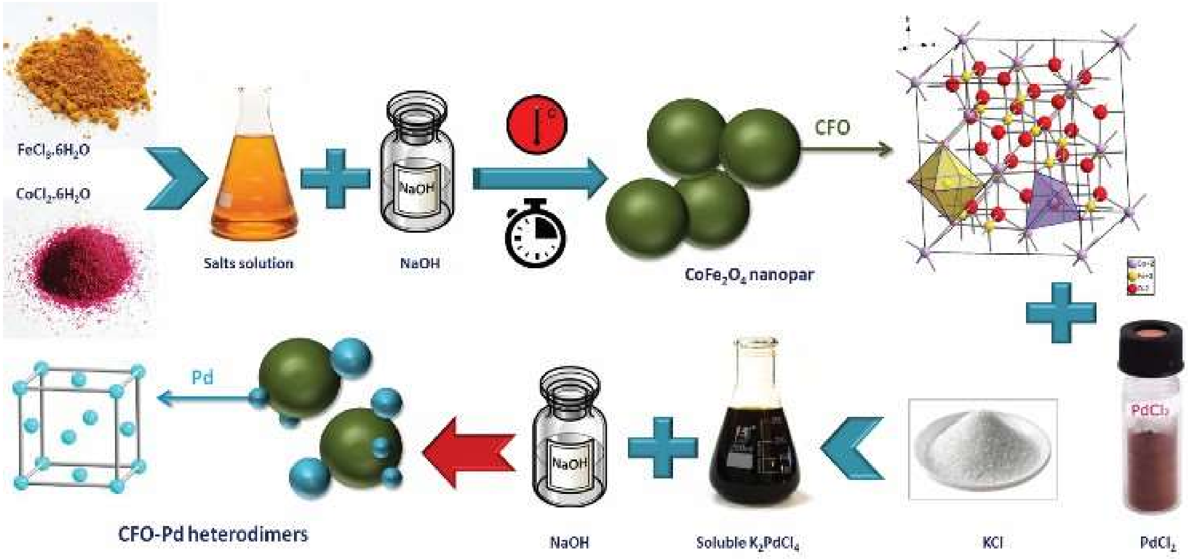

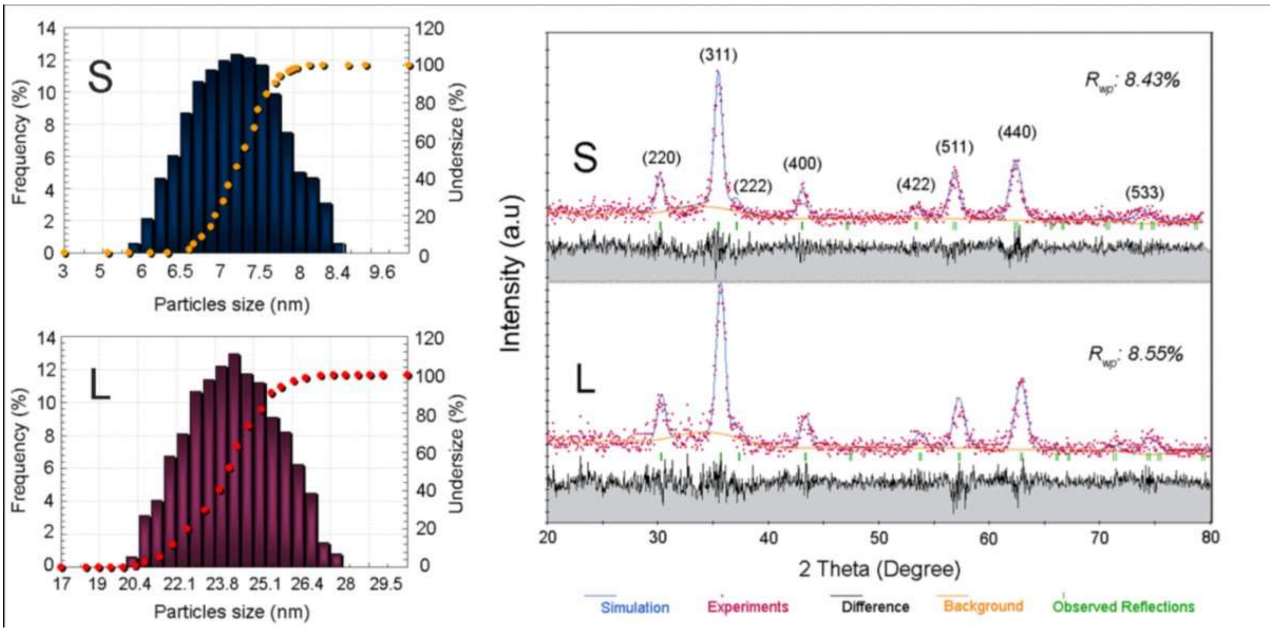

Figure S1: Distributions of diameters determined from dynamic light scattering (left); x-ray diffraction patterns and corresponding Rietveld structure refinement details (right) of small (S) and large (L) bare CFO nanoparticles.

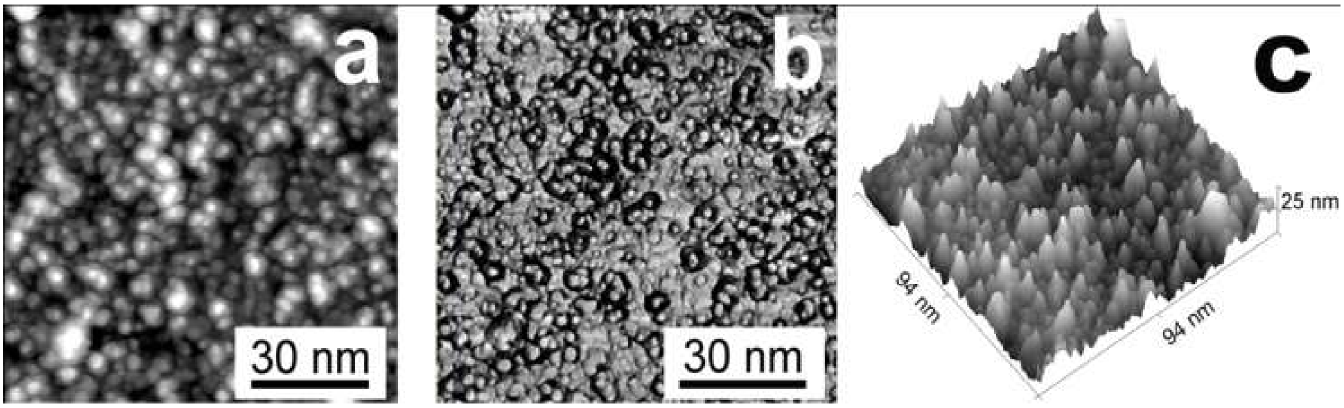

Figure S2: AFM microscopic micrographs of bare CFO nanoparticles which show the uniform particles size and morphology. (a) AFM image in topography mode. (b) AFM image in phase mode. (c) 3-D pattern.

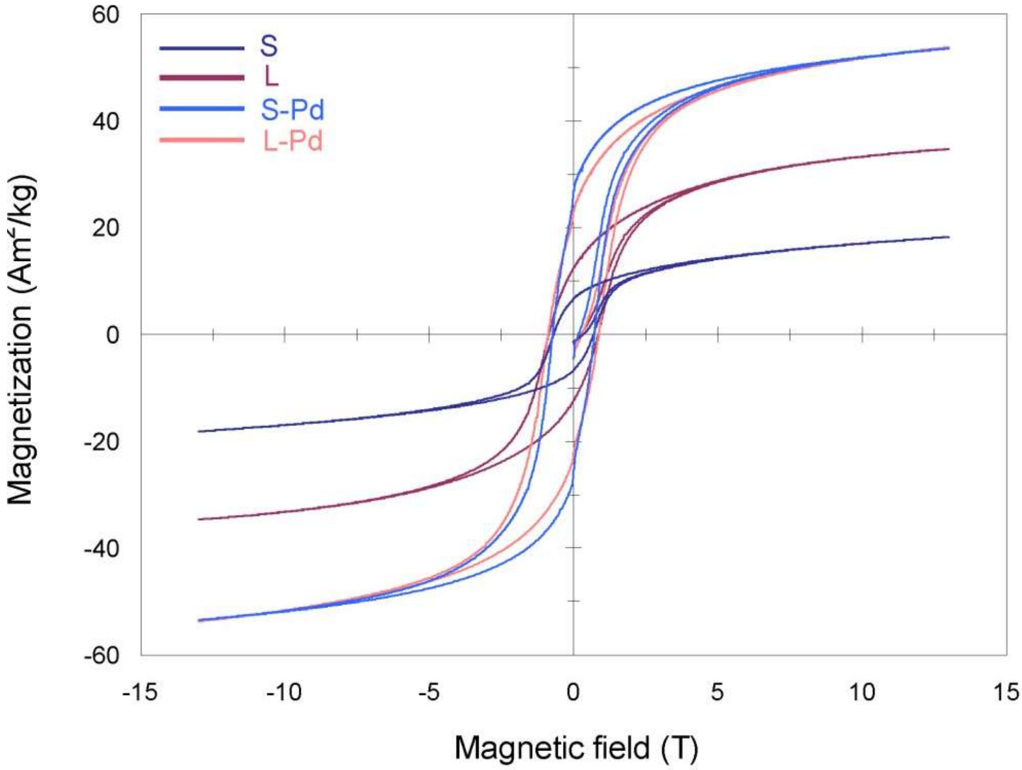

Figure S3: Field-dependent mass magnetization of bare and Pd decorated CFO nanoparticles at low temperature (8 K).

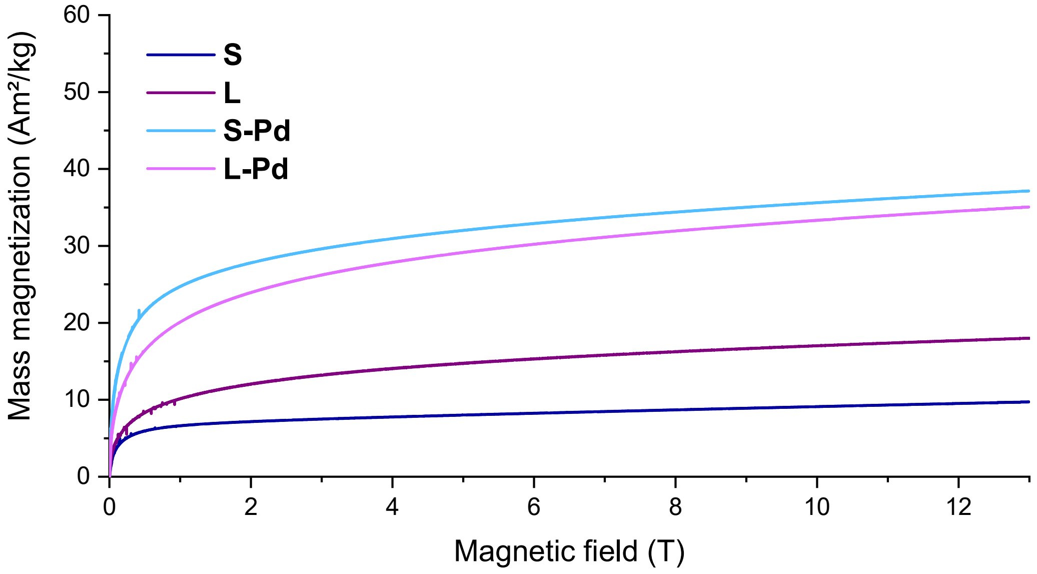

Figure S4: Field-dependent mass magnetization of bare and Pd decorated CFO nanoparticles at T = 300 K.

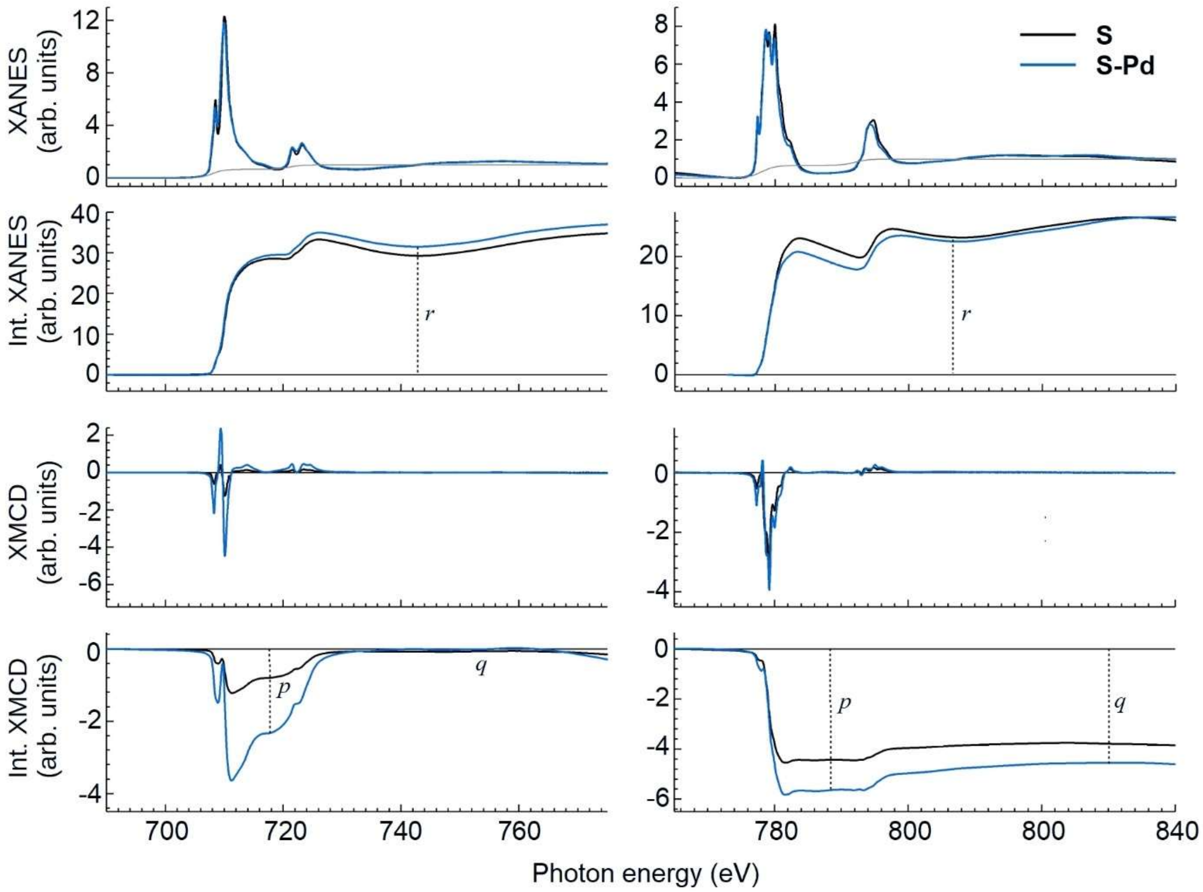

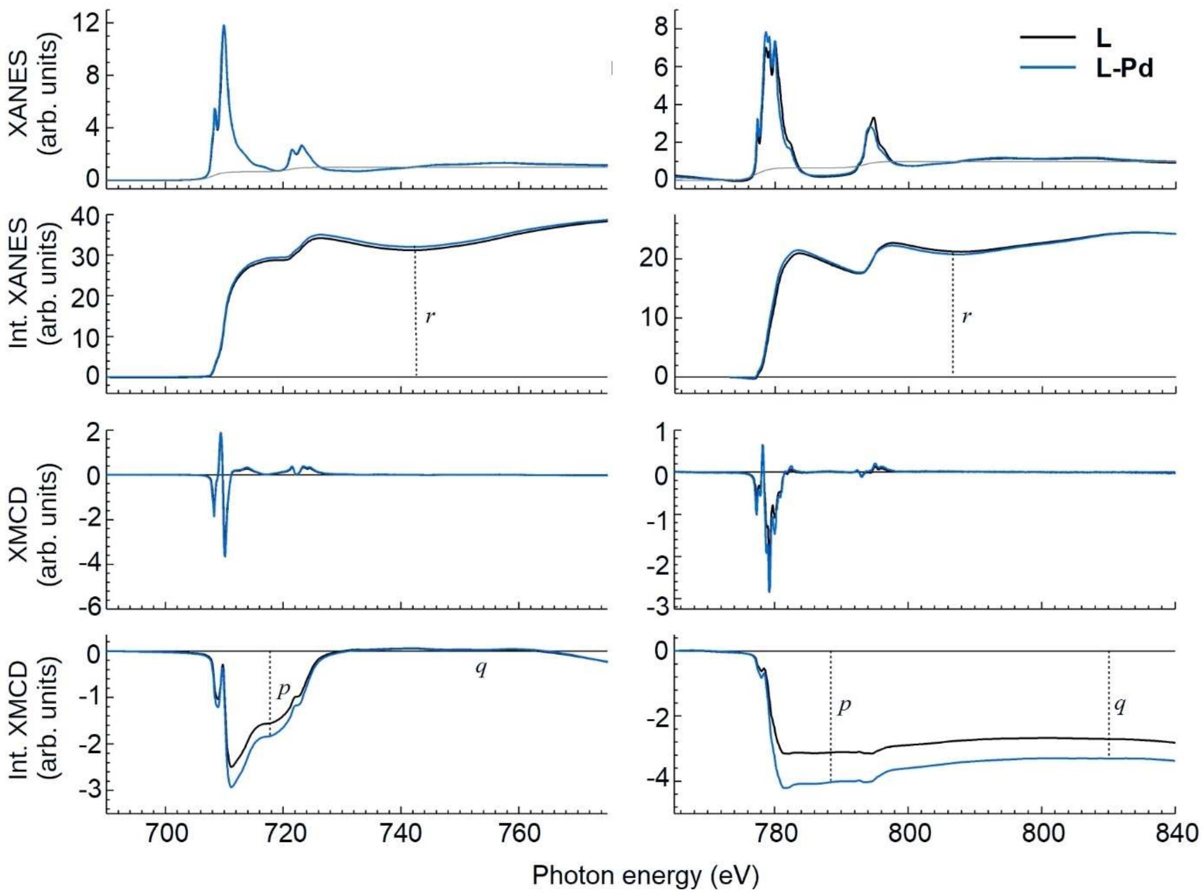

Figure S5: XANES, XMCD and their integrals for all samples at a temperature of 5 K and in a magnetic field of 6 T (UE46-PGM1).

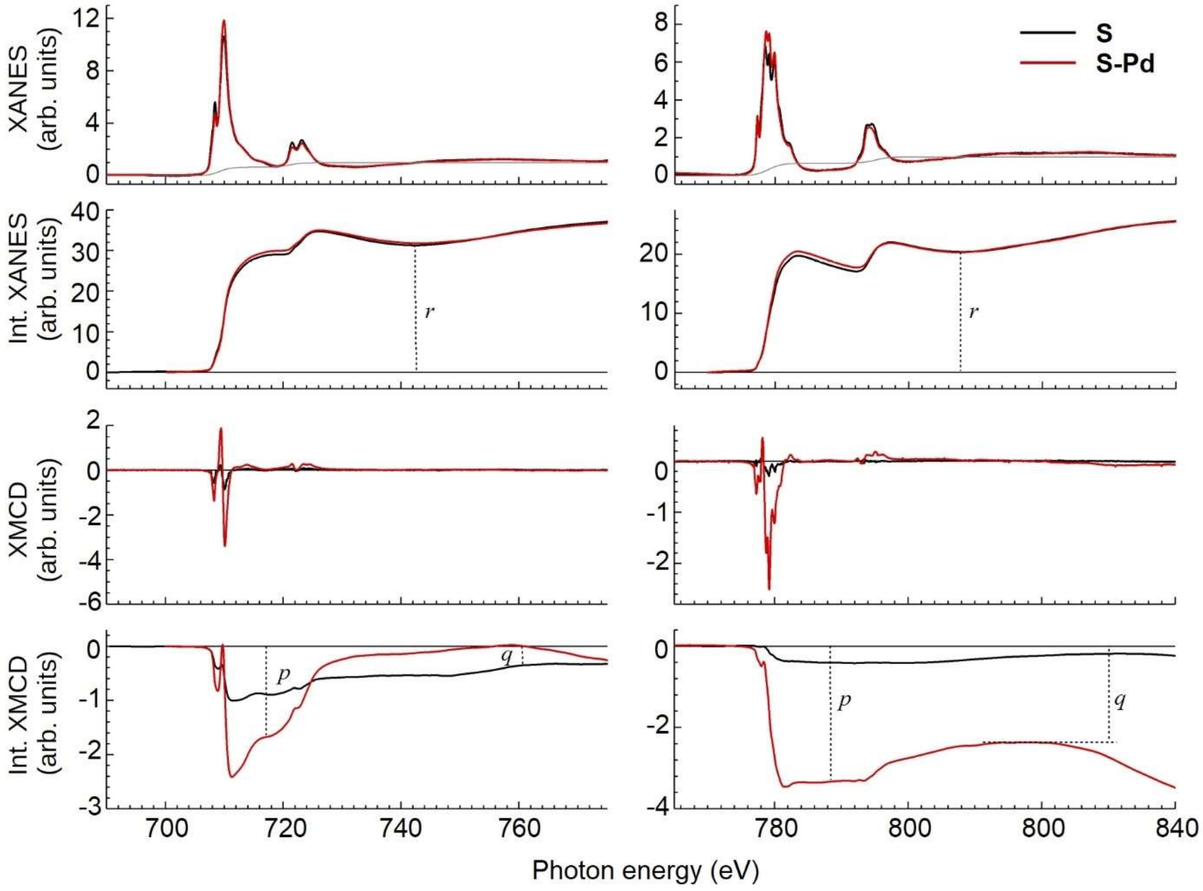

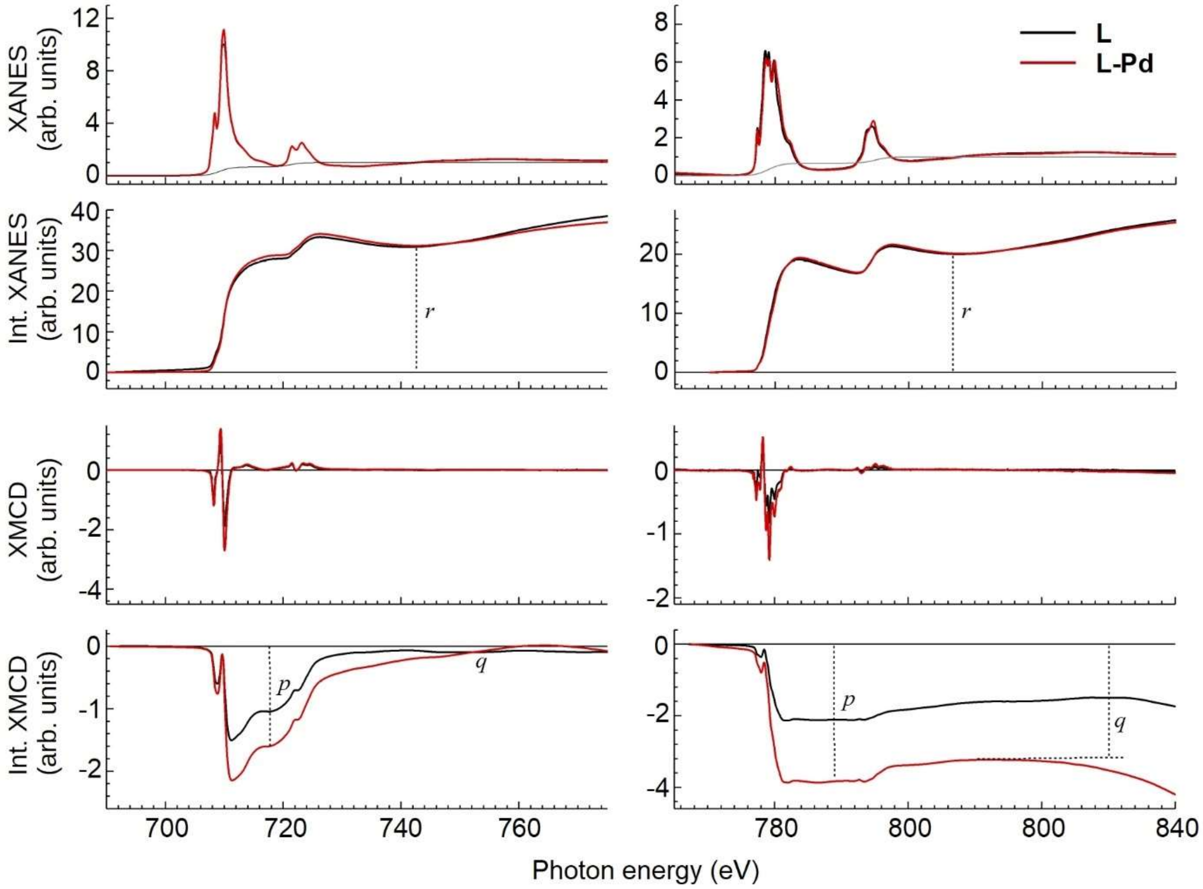

Figure S6: XANES, XMCD and their integrals for all samples at a temperature of 300 K and in a magnetic field of 6 T (UE46-PGM1).

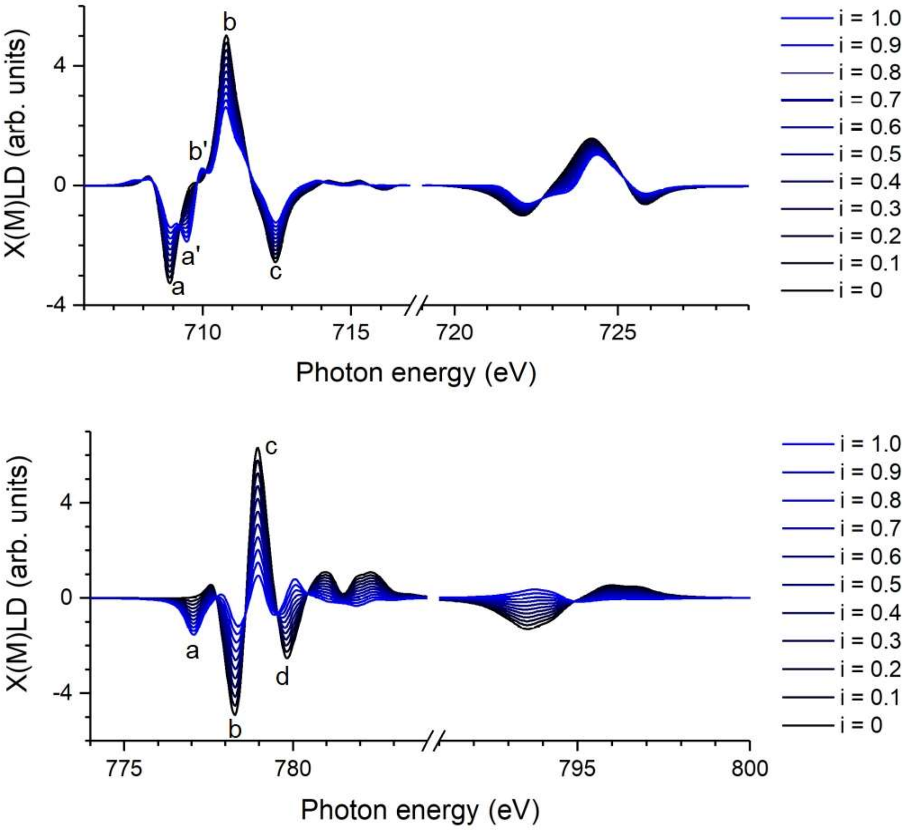

Figure S7: Simulated XMLD spectral changes with degree of inversion for Fe^{3+} (top) and Co^{2+} (bottom).

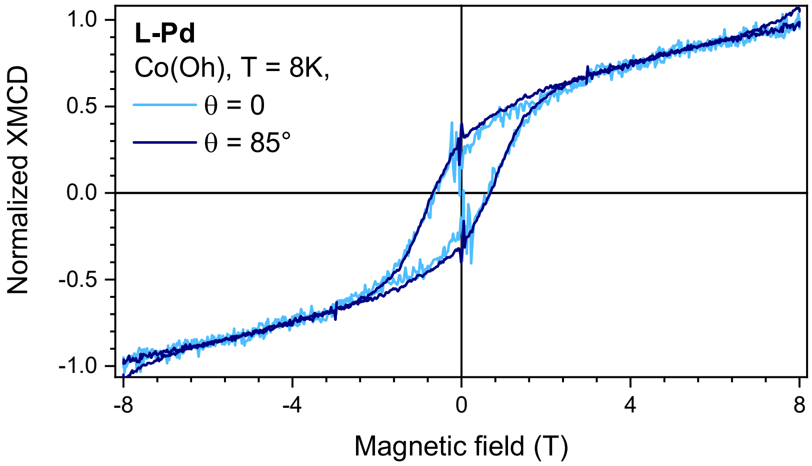

Figure S8: Field-dependent normalized XMCD for two different angles of magnetic field and incident x-rays, i.e. along the sample normal (θ = 0) and close to in-plane (θ = 85°). Data are exemplarily shown for the large CFO-Pd heterodimers at low temperature (T = 8K) measured at the Co L_3 absorption edge.

Supplementary References

[S1] A. Tomitaka, T. Koshi, S. Hatsugai, T. Yamada, Y. Takemura, Magnetic characterization of surface-coated magnetic nanoparticles for biomedical application, J. Magn. Magn. Mater., 2011, 323 (10), 1398-1403.

[S2] F. Macdonald, C. H. J. Ford, A. G. Casson, Molecular biology of cancer, BIOS Scientific Publishers, 2004.

[S3] G. F. Baronzio, E. D. Hager, Hyperthermia in cancer treatment: a primer, Springer, 2006.

[S4] E. Senkus, F. Cardoso, O. Pagani, Time for more optimism in metastatic breast cancer?, Cancer Treat. Rev., 2014, 40 (2), 220-298.

[S5] S. Wen, Y. Niu, S. O. Lee, C. Chang, Androgen receptor (AR) positive *vs* negative roles in prostate cancer cell deaths including apoptosis, anoikis, entosis, necrosis and autophagic cell death, Cancer Treat. Rev., 2014, 40 (1), 31-40.

[S6] S. Hector, J. H. M. Prehen, Apoptosis signaling proteins as prognostic biomarkers in colorectal cancer: A review, Biochimica et Biophysica Acta (BBA) - Reviews on Cancer, 2009, 1795 (2), 117-129.

[S7] P. H. Chan, B. Ghosh, H. Z. lai, H. L. Peng, K. K. T. Mong, Y. C. Chen, Photoluminescent Gold Nanoclusters as Sensing Probes for Uropathogenic Escherichia coli, PLoS ONE, 2013, 8 (3), e58064.

[S8] H. Salehizadeh, E. Hekmatian, M. Sadeghi, K. Kennedy, Synthesis and characterization of core-shell Fe3O4-gold-chitosan nanostructure, J. Nanobiotechnol., 2012, 10 (3), 1-7.

[S9] F. Mohapatra, F. Zheng, K. Elkins, M. Xing, M. Ghimire, S. Yoon, S. R. Mishra, J. P. Liu, Size-Dependent Magnetic and Inductive Heating of Fe_3O_4 Nanoparticles: Scaling Laws Across the Superparamagnetic Size, Phys. Chem. Chem. Phys., 2018, 20, 12879.
